# Microglia-targeted *Panax notoginseng* polysaccharide nanoparticles alleviate Alzheimer's disease via AMPK-mTOR/HIF-1α-mediated immunometabolic reprogramming

**DOI:** 10.1016/j.mtbio.2026.103620

**Published:** 2026-08-28

**Authors:** Ge Zhang, Liang Kong, Rui-bo Guo, Shuai-wen Ding, Yang Liu, Juan Zang, Ying Zheng, Bin Wei, Zhi-chao Chen, Ying Yang, Xue-tao Li, Yang Yu

**Affiliations:** a College of Pharmacy, Liaoning University of Traditional Chinese Medicine, Dalian, 116600, China; b Department of Biomedical Engineering, Dalian University of Technology, Dalian, 116024, China; c Affiliated Hospital of Liaoning University of Traditional Chinese Medicine, Shenyang, 110847, China; d Shenyang Key Laboratory of Chinese Medicine Targeted Delivery, Shenyang, 110148, China

**Keywords:** Alzheimer's disease, Immunometabolism, *Panax notoginseng* polysaccharide, Microglia, AMPK/mTOR/HIF-1α, Metabolic reprogramming, Biomaterials

## Abstract

Alzheimer's disease (AD) is increasingly recognized as an immunometabolic disorder characterized by a self-reinforcing cycle of neuroinflammation and glycolytic reprogramming in microglia. Herein, we developed KPBIT@NPs, a microglia-targeted polysaccharide-based nanomedicine. Unlike many conventional nanocarriers that mainly serve as passive delivery vehicles, KPBIT@NPs employ the natural macromolecule *Panax notoginseng* polysaccharide (PNP) to construct a dual-functional, responsive nanoparticle platform. Through rational engineering via phenylboronic ester grafting, the polysaccharide backbone enables amphiphilic self-assembly and efficient co-loading of icaritin and tanshinone IIA, while allowing pathological reactive oxygen species (ROS)-triggered disassembly. In addition, surface decoration with the KLVFFAED peptide facilitates blood–brain barrier transport and targeted accumulation in activated microglia. Mechanistically, KPBIT@NPs activate AMP-activated protein kinase and suppress the mechanistic target of rapamycin/hypoxia-inducible factor 1α signaling axis, thereby reversing pathological glycolytic skewing and restoring mitochondrial oxidative metabolism. This coordinated regulation disrupts the inflammation–metabolism feed-forward loop and successfully reprograms microglia from a pro-inflammatory, metabolically stressed state toward a reparative phenotype. In AD animal models, KPBIT@NPs effectively restore cerebral energy metabolism, markedly reduce amyloid-β deposition, alleviate neuroinflammation, preserve neuronal integrity, and ultimately rescue cognitive deficits. This study establishes an innovative nanotherapeutic paradigm based on bioactive polysaccharides and offers a promising materials strategy for neurodegenerative diseases driven by coupled metabolic and inflammatory dysregulation.

## Highlights

• A multifunctional *Panax notoginseng* polysaccharide nanoplatform is developed for brain-targeted co-delivery of icaritin and tanshinone IIA.
• KPBIT@NPs reprogram microglial immunometabolism through coordinated regulation of the AMPK-mTOR/HIF-1α axis.
• The formulation shifts microglia from the pro-inflammatory M1 phenotype to the anti-inflammatory M2 phenotype and alleviates neuroinflammation.

## Graphical abstract

Alzheimer's disease (AD) is increasingly recognized as an immunometabolic disorder in which microglia become trapped in a self-amplifying inflammatory and glycolytic state. However, most current therapeutic strategies either target amyloid pathology or suppress inflammation broadly, while failing to precisely reprogram the metabolic circuitry that sustains microglial dysfunction. Here, we develop a microglia-targeted, reactive oxygen species-responsive polysaccharide nanomedicine, KPBIT@NPs, that functions as a biomaterial-enabled immunometabolic regulator for AD therapy. Unlike conventional inert nanocarriers, KPBIT@NPs are constructed from *Panax notoginseng* polysaccharide, a bioactive natural macromolecule with intrinsic immunomodulatory potential, and are rationally engineered through phenylboronic ester grafting to achieve amphiphilic self-assembly, pathological ROS-triggered disassembly, and efficient co-loading of icaritin and tanshinone IIA. Surface decoration with the KLVFFAED peptide enables blood–brain barrier transport and preferential accumulation in activated microglia, thereby establishing a hierarchical delivery cascade from brain entry to disease-cell-specific intervention. Mechanistically, KPBIT@NPs act on a central microglial energy checkpoint by restoring AMPK activity and suppressing the mTOR/HIF-1α axis. This coordinated regulation reverses the pathological glycolytic switch, rescues mitochondrial oxidative metabolism, and interrupts the inflammation–metabolism feed-forward loop that drives chronic neuroinflammation. As a result, microglia are reprogrammed from a pro-inflammatory, metabolically stressed state toward a reparative phenotype with enhanced mitochondrial function and reduced inflammatory output. In AD models, KPBIT@NPs significantly attenuate amyloid-β burden, mitigate neuroinflammation, preserve neuronal and mitochondrial integrity, and improve cognitive performance. Further biochemical assessment demonstrates recovery of ATP production and tricarboxylic acid cycle-related metabolic activity, providing functional evidence that KPBIT@NPs alleviate AD-associated cerebral energy metabolism impairment. This study establishes a polysaccharide-based nanotherapeutic paradigm for microglial immunometabolic reprogramming and provides a generalizable materials strategy for treating neurodegenerative diseases driven by coupled metabolic and inflammatory dysregulation.

**Figure:**
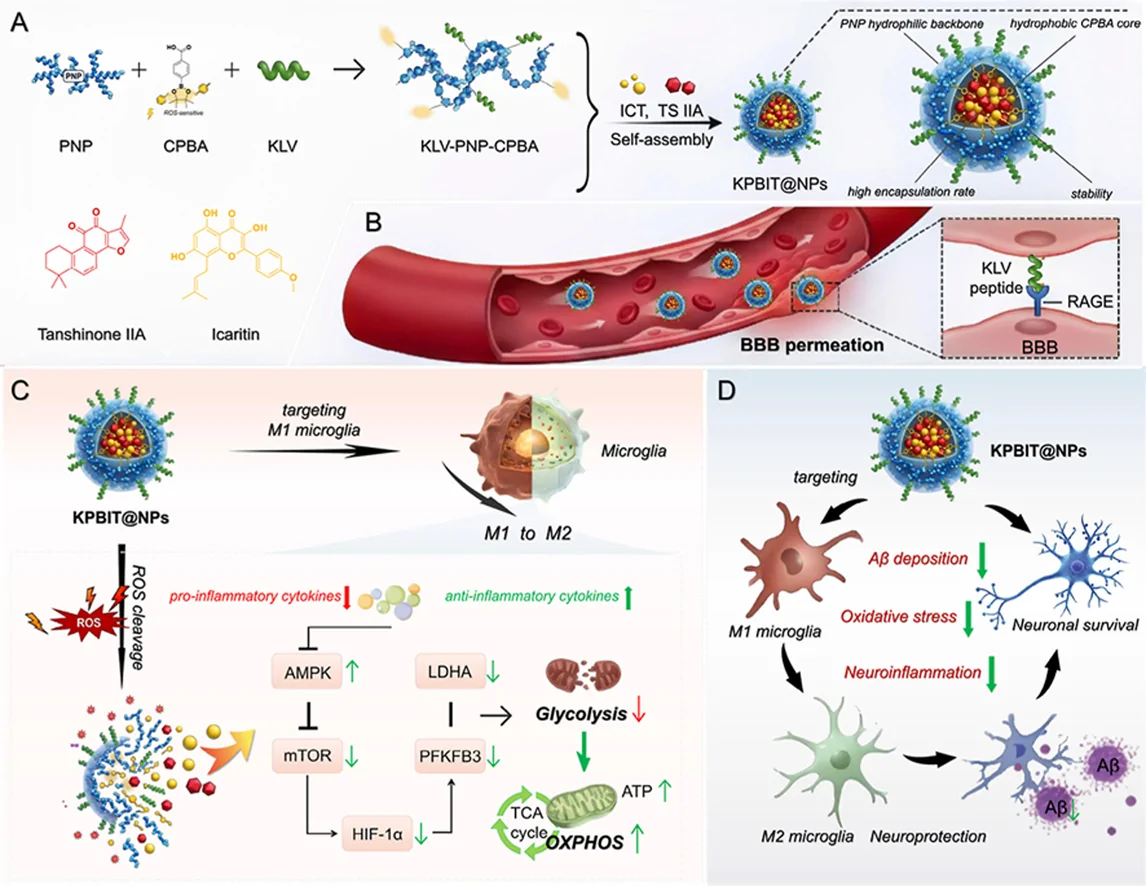

## 1 Introduction

Alzheimer's disease (AD) is a progressive neurodegenerative disorder and one of the most challenging diseases facing aging societies. Although amyloid-β (Aβ) deposition, tau pathology, synaptic dysfunction, and neuronal loss have long been considered central pathological features of AD, increasing evidence indicates that the disease cannot be fully explained by neuron-centered mechanisms alone [1]. Microglia, the resident innate immune cells of the central nervous system, actively sense, integrate, and remodel the pathological microenvironment of the AD brain. Rather than serving merely as inflammatory effector cells, microglia function as dynamic regulators of protein clearance, synaptic remodeling, mitochondrial stress, and tissue repair [[2], [3], [4]]. Once chronically activated by Aβ, oxidative stress, and persistent inflammatory signals, microglia progressively lose their homeostatic surveillance capacity and acquire maladaptive phenotypes that accelerate neurodegeneration. Therefore, strategies capable of restoring microglial homeostasis have emerged as a critical frontier for AD intervention.

A key but insufficiently addressed feature of microglial dysfunction in AD is immunometabolic reprogramming. Activated microglia undergo profound metabolic remodeling, shifting from mitochondrial oxidative phosphorylation (OXPHOS) toward aerobic glycolysis to support rapid inflammatory activation, cytokine production, and biosynthetic demand [[5], [6], [7], [8], [9]]. Although this metabolic transition may be adaptive during acute immune responses, its chronic persistence in AD becomes pathological. Glycolysis-dominant microglia exhibit impaired mitochondrial respiration, excessive reactive oxygen species (ROS) production, and amplified inflammatory signaling, collectively forming a self-reinforcing inflammation–metabolism loop. In contrast, microglia with reparative and resolution-associated functions depend more heavily on intact mitochondrial oxidative metabolism and tricarboxylic acid cycle activity [[10], [11], [12], [13]]. Thus, AD progression can be viewed, at least in part, as a failure of microglial metabolic plasticity, in which immune cells are locked into a pro-inflammatory bioenergetic state. Rewiring this dysfunctional metabolic program offers a mechanistically distinct therapeutic opportunity beyond conventional anti-amyloid or non-specific anti-inflammatory approaches.

Among the signaling networks that coordinate microglial metabolism and immune function, the AMP-activated protein kinase (AMPK)–mechanistic target of rapamycin (mTOR)–hypoxia-inducible factor 1α (HIF-1α) axis represents a central metabolic checkpoint. AMPK acts as a cellular energy sensor that preserves mitochondrial homeostasis, promotes catabolic energy generation, and restrains excessive inflammatory activation. In contrast, mTOR integrates nutrient, growth factor, and immune signals to promote anabolic metabolism and glycolytic activation [[14], [15], [16]]. Aberrant mTOR activation enhances HIF-1α signaling, which transcriptionally drives glycolytic enzymes and glucose transporters, thereby stabilizing the glycolytic and pro-inflammatory phenotype of activated microglia [17,18]. In the AD microenvironment, Aβ accumulation, oxidative stress, and chronic inflammation impair AMPK activity while sustaining mTOR/HIF-1α activation, leading to mitochondrial dysfunction and persistent inflammatory metabolic remodeling [[19], [20], [21], [22]]. Therefore, merely blocking a single inflammatory mediator is unlikely to reset microglial function. A more effective strategy should simultaneously restore AMPK-dependent energy sensing and suppress mTOR/HIF-1α-driven glycolytic programming, thereby converting microglia from inflammatory amplifiers into metabolically competent repair cells.

Natural products provide a rich chemical space for multi-target regulation of complex diseases such as AD. Traditional Chinese medicine (TCM) has long applied the principle of “BuShen HuoXue” in dementia-related disorders, with *Epimedium* and *Salvia miltiorrhiza* representing two classical medicinal resources. Their bioactive constituents, icaritin (ICT) and tanshinone IIA (TS IIA), have attracted increasing attention owing to their neuroprotective, anti-inflammatory, antioxidant, and microglia-modulating activities [[23], [24], [25], [26], [27], [28]]. Distinct from conventional broad-spectrum anti-inflammatory compounds, the combination of these two agents may provide mechanistically complementary effects. ICT has been reported to modulate cellular energy sensing and mitochondrial homeostasis [[29], [30], [31]], whereas TS IIA has shown particular potential in suppressing neuroinflammation and metabolic stress and can restrain glycolysis-driven pro-inflammatory programs [[32], [33], [34]]. Importantly, previous biological studies and network pharmacology analyses suggest that these two compounds may converge on immune and metabolic pathways, including AMPK and mTOR signaling, making them promising candidates for coordinated immunometabolic intervention. However, their therapeutic potential is severely constrained by poor aqueous solubility, limited brain accumulation, suboptimal pharmacokinetics, and insufficient delivery to disease-relevant microglia [35,36]. These limitations highlight the need for a biomaterial platform capable of transforming multi-component natural products into a precise, brain-accessible, cell-targeted, and microenvironment-responsive therapeutic system.

Nanomedicine offers important opportunities to overcome the multiple biological barriers that limit AD therapy, including blood–brain barrier (BBB) restriction, heterogeneous lesion distribution, and insufficient intracellular drug availability [[37], [38], [39], [40], [41]]. In recent years, BBB-targeted and microglia-targeted nanosystems have made substantial progress in AD treatment. Through ligand functionalization, peptide-mediated transport, cell membrane coating, exosome/extracellular vesicle-mimetic delivery, and stimuli-responsive material design, previous studies have improved brain delivery, lesion accumulation, and disease-relevant cellular uptake of nanomedicines, showing promise in promoting Aβ clearance, alleviating oxidative stress, suppressing neuroinflammation, and enhancing neuroprotection [[42], [43], [44]]. However, most existing strategies primarily aim to improve delivery efficiency or achieve broad anti-inflammatory and antioxidant effects, while few are mechanistically designed to correct the immunometabolic imbalance of activated microglia in AD. In particular, nanoplatforms capable of simultaneously achieving BBB penetration, activated microglia recognition, pathological ROS-responsive release, and regulation of the AMPK-mTOR/HIF-1α metabolic checkpoint remain scarce. More importantly, many nanocarriers still function mainly as drug transport vehicles, with their material backbones contributing only minimally to the regulation of the AD microenvironment. Therefore, constructing a nanoplatform that combines precise delivery capability with intrinsic disease-modulating activity represents a critical challenge for advancing immunometabolic therapy for AD.

In this context, polysaccharide-based biomaterials possess unique advantages. Compared with inert synthetic carriers, polysaccharides exhibit favorable biocompatibility, biodegradability, abundant chemical modification sites, and potential immunomodulatory activity. Thus, they can serve not only as drug delivery scaffolds but also as functional components that participate in the regulation of disease microenvironments. *Panax notoginseng* polysaccharide (PNP) is particularly attractive because of its antioxidant, anti-inflammatory, and immunomodulatory properties, as well as its structural plasticity, which allows chemical engineering to introduce self-assembly, targeted recognition, and stimuli-responsive functions [45]. Therefore, constructing bioactive nanocarriers from PNP may overcome the transport-centered limitations of conventional nanodelivery systems and enable the material backbone itself to become a functional element in AD therapeutic design.

**Scheme 1:**
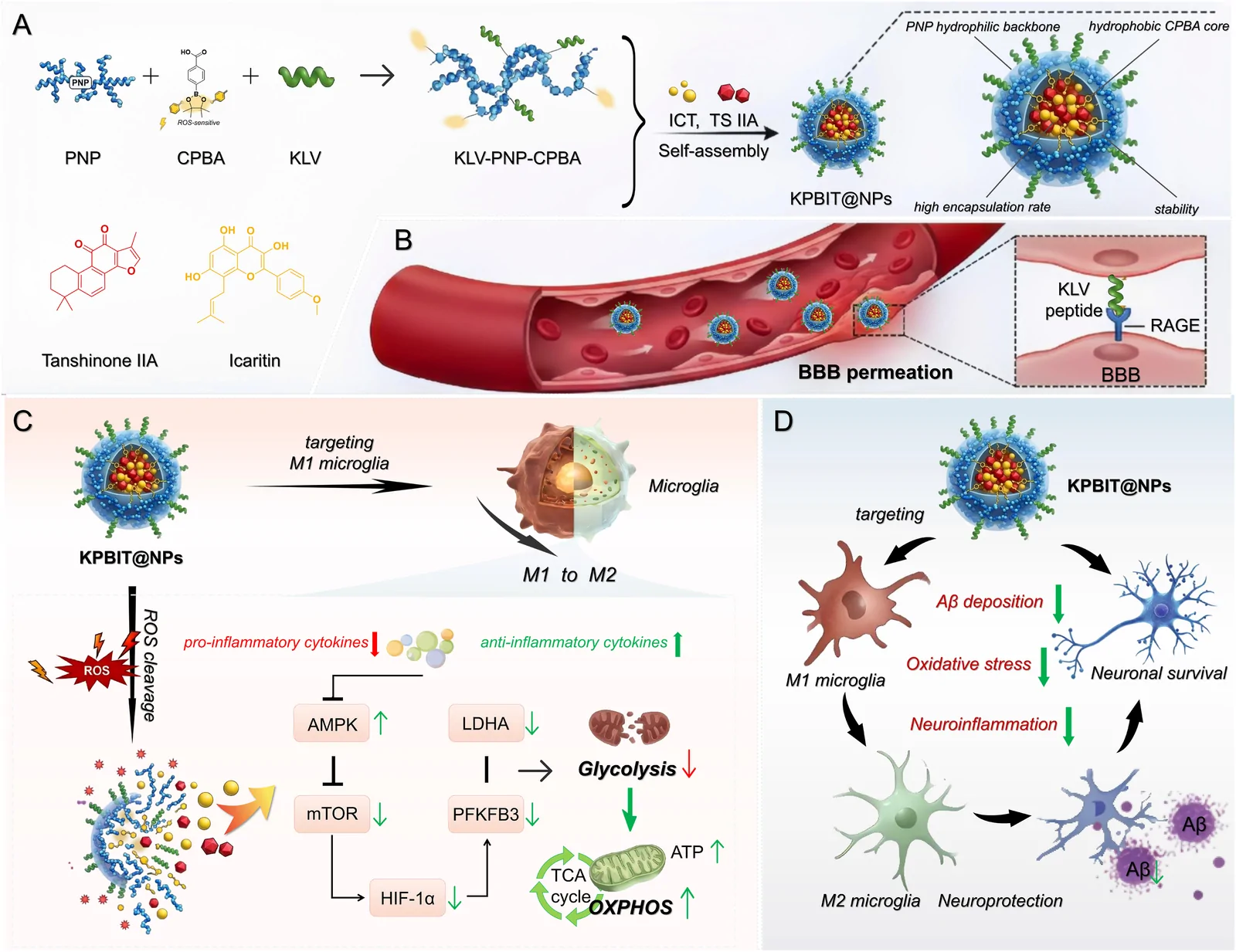
**Schematic illustration of the construction and anti-AD mechanisms of KPBIT@NPs.** (A) Construction process of KPBIT@NPs. (B) Schematic illustration of the mechanism by which KPBIT@NPs cross the BBB. (C) Schematic illustration of the mechanism by which KPBIT@NPs promote the polarization of microglia from the M1 phenotype to the M2 phenotype and improve the brain microenvironment through regulation of the AMPK-mTOR/HIF-1α pathway. (D) Schematic illustration of the anti-AD mechanism of KPBIT@NPs mediated by microglial polarization.

In our previous study, we identified a PNP with favorable self-assembly potential and biocompatibility, providing a material basis for the construction of a bioactive nanodelivery system [46]. Building on this foundation, we developed KLVFFAED- and 4-carboxyphenylboronic acid pinacol ester (CPBA)-co-modified *Panax notoginseng* polysaccharide nanoparticles co-loaded with ICT and TS IIA (KPBIT@NPs), a multifunctional PNP-based nanoplatform designed to address microglial immunometabolic abnormalities in AD. In this system, CPBA was grafted onto the PNP backbone to introduce amphiphilicity and ROS-sensitive phenylboronic ester bonds, thereby enabling nanoparticle self-assembly and responsive drug release in the oxidative pathological microenvironment. ICT and TS IIA were co-loaded to synergistically intervene in the aberrant coupling between microglial energy metabolism and inflammatory activation, while the KLVFFAED (KLV) peptide was further incorporated to enhance BBB crossing and selective uptake by activated microglia. This study proposes a bioactive polysaccharide-based nanotherapeutic strategy for AD and elucidates how natural macromolecular biomaterials can be engineered to achieve disease-cell-specific immunometabolic reprogramming. Beyond AD, this strategy provides a generalizable framework for treating neurodegenerative and neuroinflammatory diseases jointly driven by metabolic and immune dysfunction (Scheme 1).

## 2 Results and discussion

### 2.1 Construction and structural characterization of amphiphilic polysaccharide derivatives

**Fig. 1:**
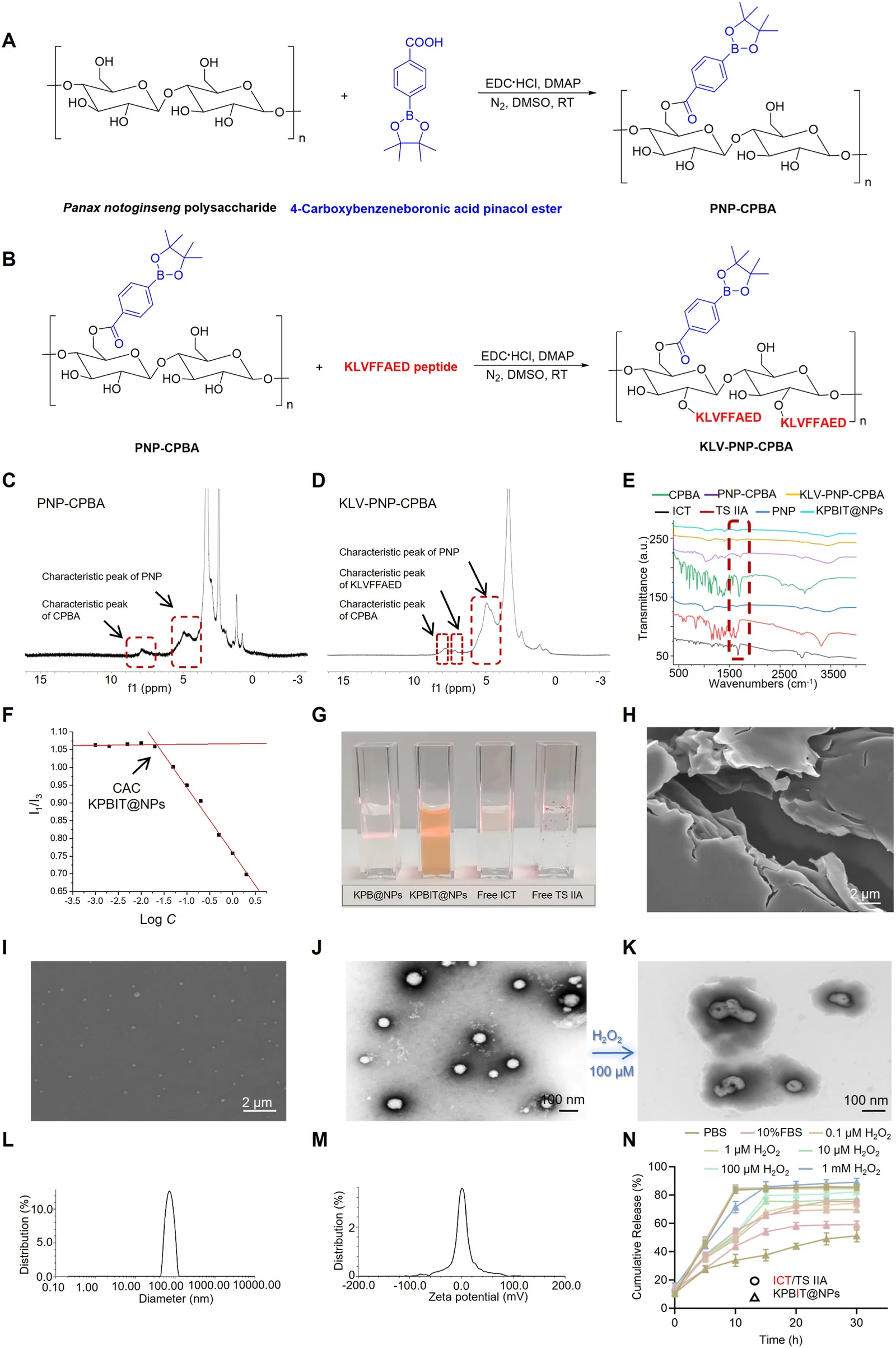
**Synthesis and physicochemical characterization of ROS-responsive nanofor**mulations. (A) Synthetic route of PNP-CPBA. (B) Synthetic route of KLV-PNP-CPBA. (C) ^1^H NMR spectrum of PNP-CPBA. (D) ^1^H NMR spectrum of KLV-PNP-CPBA. (E) FTIR spectra of raw materials, free drugs, and drug-loaded formulations. (F) CAC of KPBIT@NPs. (G) Photographs of KPB@NPs, KPBIT@NPs, free ICT, and free TS IIA in aqueous solution. (H) SEM image of PNP, Scale bar, 2 μm. (I) SEM image of KPBIT@NPs, Scale bar, 2 μm. (J) TEM image of KPBIT@NPs, Scale bar, 100 nm. (K) TEM image of KPBIT@NPs after the nanoparticles were incubated in PBS, pH 7.4 containing 100 μM H_2_O_2_ at room temperature for 24 h oxidative treatment, Scale bar, 100 nm. (L) Particle size distribution of KPBIT@NPs. (M) Zeta potential of KPBIT@NPs. (N) Cumulative release profile of ICT from KPBIT@NPs in PBS, 10% FBS, and H_2_O_2_ solutions (0.1-100 μM and 1 mM) (n = 3).

To develop a multifunctional nanocarrier with self-assembly capability, responsiveness to disease-associated microenvironments, and targeting functionality, PNP was employed as the backbone. CPBA was grafted to introduce hydrophobic domains. Subsequently, the KLV was covalently conjugated, yielding a peptide-functionalized amphiphilic polysaccharide (Fig. 1A and B). proton nuclear magnetic resonance (^1^H NMR) spectra confirmed the successful grafting of CPBA onto the polysaccharide backbone. Characteristic proton signals of the polysaccharide appeared at *δ* 3.0–5.5 ppm, while aromatic proton signals from CPBA were observed at *δ* 7.5–8.0 ppm (Fig. 1C). After peptide conjugation, additional signals attributed to aromatic residues of the peptide emerged at *δ* 7.0–7.5 ppm, while the original signals from PNP and CPBA were retained. These results suggest that the backbone structure was largely preserved during functionalization, indicating a stepwise and controlled modification process (Fig. 1D). Fourier transform infrared (FTIR) spectroscopy further verified the successful construction of the material. Compared with native PNP, PNP-CPBA exhibited a characteristic ester carbonyl absorption peak at ∼1730 cm^−1^, indicating the covalent incorporation of hydrophobic groups into the polysaccharide chain (Fig. 1E). Together, these findings confirm the successful construction of amphiphilic polysaccharide derivatives, providing a robust material foundation for subsequent self-assembly and nanoparticle fabrication.

### 2.2 CPBA grafting density-regulated self-assembly behavior and structure–property relationships

The self-assembly behavior of amphiphilic polymers is highly dependent on the hydrophilic–hydrophobic balance. To systematically investigate the effect of CPBA grafting density on the self-assembly of PNP-CPBA, a series of derivatives were prepared by varying the CPBA feed ratio. Their grafting degree, critical aggregation concentration (CAC), hydrodynamic diameter, and polydispersity index (PDI) were systematically quantified and analyzed (Supplementary Table S1). As the CPBA feed increased from 0.01 mmol to 1.0 mmol, the grafting degree rose from 0.87% to 13.61%, demonstrating a controllable structure–composition relationship. Meanwhile, as the grafting degree increased from 0.87% to 8.95%, the CAC decreased markedly from 0.697 mg/mL to 0.0358 mg/mL. This trend suggests that increasing hydrophobic density enhances intermolecular hydrophobic interactions, enabling the system to overcome steric and hydration repulsion and spontaneously form stable nanoparticles at lower concentrations. Notably, when the CPBA feed was further increased to 0.75 mmol and 1.0 mmol (corresponding to grafting degrees of 11.73% and 13.61%, respectively), excessive hydrophobic substitution disrupted the hydrophilic–hydrophobic balance. This resulted in reduced aqueous solubility and poor dispersion, leading to aggregation instability. These findings highlight a non-linear dependence of self-assembly behavior on grafting density, indicating that hydrophobic modification promotes self-assembly only within an optimal range. Based on these results, PNP-CPBA with a grafting degree of 8.95% was selected for subsequent studies, as it exhibited the lowest CAC while maintaining good aqueous solubility, representing an optimal balance between colloidal stability and assembly efficiency.

### 2.3 Construction, physicochemical characterization, and encapsulation efficiency of KPBIT@NPs

Based on the optimized amphiphilic polysaccharide derivative, peptide-modified dual drug-loaded nanoparticles (KPBIT@NPs) were constructed via a co-assembly strategy to simultaneously encapsulate ICT and TS IIA. The CAC, determined using pyrene as a fluorescent probe, was 0.0216 mg/mL (Fig. 1F). The relatively low CAC indicates a favorable tendency of the amphiphilic polysaccharide derivative to maintain self-assembled structures upon dilution under simplified equilibrium conditions. However, CAC alone cannot directly predict nanoparticle integrity in the dynamic blood environment, where rapid dilution, plasma proteins, lipoproteins, electrolytes, and cellular components may alter the self-assembly equilibrium and colloidal behavior. In contrast to free ICT and TS IIA, which exhibit poor aqueous dispersibility with visible precipitation, KPBIT@NPs formed a clear and stable orange solution, suggesting that nanoencapsulation significantly improves the apparent solubility of both hydrophobic drugs (Fig. 1G). The Tyndall effect further confirmed the formation of micellar nanoparticles. FTIR analysis showed that the characteristic absorption peaks of ICT and TS IIA were absent in KPBIT@NPs, which is consistent with their encapsulation within the hydrophobic core (Fig. 1E). The encapsulation efficiencies were (92.10 ± 2.09)% for ICT and (87.49 ± 1.93)% for TS IIA (Supplementary Table S2), indicating high encapsulation efficiency. Scanning electron microscopy (SEM) and transmission electron microscopy (TEM) images acquired from multiple representative fields revealed that KPBIT@NPs formed well-dispersed nanoparticles with a relatively uniform, quasi-spherical morphology (Fig. 1I, J and Supplementary Fig. S1A and B), whereas native *Panax notoginseng* polysaccharide exhibited an irregular amorphous structure (Fig. 1H). Dynamic light scattering (DLS) analysis showed that the blank carrier KPB@NPs had a hydrodynamic diameter of 66.320 ± 0.826 nm with a PDI of 0.2160 ± 0.0304, while the non-targeted drug-loaded nanoparticles PBIT@NPs exhibited a diameter of 76.390 ± 0.717 nm and a PDI of 0.1840 ± 0.0951 (Supplementary Table S3). The final formulation, KPBIT@NPs, showed a slightly larger hydrodynamic diameter of 79.330 ± 0.191 nm (Fig. 1L), a lower PDI of 0.0593 ± 0.0442, and a near-neutral zeta potential of −2.10 ± 0.55 mV (Fig. 1M), indicating good size uniformity and colloidal dispersibility. The near-neutral surface charge, together with the hydrophilic polysaccharide shell, may contribute to reduced nonspecific protein adsorption and steric stabilization.

The colloidal stability of KPBIT@NPs was further evaluated under physiologically relevant conditions, including storage at 4°C and incubation in 10% fetal bovine serum (FBS). Over 14 days, the particle size increased gradually from approximately 79 to 90 nm at 4°C and from 83 to 100 nm in 10% FBS, corresponding to total increases of 13.5% and 20.6%, respectively. No abrupt aggregation was observed under either condition, and the PDI values remained below 0.24 throughout the observation period (Supplementary Table S4), suggesting that KPBIT@NPs maintained favorable colloidal stability and structural integrity under the tested in vitro conditions.

Nevertheless, these simplified in vitro systems cannot fully recapitulate the complexity of plasma or whole blood. After intravenous administration, nanoparticles rapidly interact with albumin, immunoglobulins, complement proteins, apolipoproteins, fibrinogen, and other plasma components, leading to the formation of a dynamic protein corona. The composition and thickness of this corona are influenced by nanoparticle size, surface charge, surface chemistry, protein concentration, nanoparticle-to-protein ratio, incubation time, and the biological medium used [47,48]. Therefore, stability assays performed in 10% FBS are useful for preliminary screening but may underestimate protein adsorption and corona formation under full-plasma or whole-blood conditions.

Protein corona formation may either destabilize or stabilize nanoparticles, depending on the composition and organization of the adsorbed proteins. On one hand, protein adsorption can promote interparticle bridging, surface-charge screening, hydrophobic interactions, aggregation, increased hydrodynamic diameter and PDI, masking of the KLV peptide, altered cellular recognition, or premature drug leakage. On the other hand, a relatively uniform protein coating may enhance colloidal stability through steric stabilization or surface passivation [[49], [50], [51], [52]]. Collectively, the low CAC, favorable serum stability, and sustained drug-release profiles demonstrate that KPBIT@NPs possess stable self-assembly behavior and effective cargo-retention capacity under the tested in vitro conditions. These results provide important evidence supporting the structural robustness of KPBIT@NPs and their suitability for subsequent biological evaluation.

### 2.4 ROS-responsive structural disassembly and controlled drug release behavior

In addition to colloidal stability, ROS responsiveness is an important feature for achieving pathology-adaptive drug release. TEM observation showed that KPBIT@NPs underwent marked morphological disruption and fragmentation after incubation with H_2_O_2_, suggesting that oxidative cleavage of phenylboronic ester bonds promoted nanoparticle disassembly (Fig. 1K). This ROS-responsive structural change was further supported by the in vitro release profiles.

Free ICT and TS IIA were rapidly released through dissolution and diffusion, reaching a plateau within a short time because no carrier-mediated retention barrier was present. By contrast, KPBIT@NPs exhibited minimal premature drug release in phosphate-buffered saline (PBS) without H_2_O_2_ and maintained a sustained release profile, confirming efficient payload retention and good structural stability under non-oxidative conditions. In 10% FBS, KPBIT@NPs displayed a similar sustained-release pattern, with only a modest increase in release rate compared with PBS, likely owing to serum protein adsorption and slight perturbation of the nanoparticle surface or core structure.

In sharp contrast, exposure to H_2_O_2_ markedly accelerated drug release from KPBIT@NPs, demonstrating clear ROS-triggered release behavior (Fig. 1N and Supplementary Fig. S2). The H_2_O_2_ concentration gradient of 0.1 μM to 1 mM was selected based on previously reported ROS-responsive drug-delivery systems and the concentration-dependent biological effects of H_2_O_2_, allowing evaluation of the formulation response from mild oxidative stimulation to high oxidative challenge [53,54]. Specifically, 0.1–10 μM H_2_O_2_ was used to assess the sensitivity of KPBIT@NPs to relatively mild oxidative stimulation, whereas 100 μM H_2_O_2_ represented a stronger oxidative challenge commonly used in in vitro ROS-responsive material evaluation. The 1 mM H_2_O_2_ condition was included as a high-oxidative-stress control to verify extensive phenylboronic ester cleavage and define the upper release response of KPBIT@NPs. This concentration series was therefore designed to characterize the material response across a broad oxidative range rather than to directly reproduce the transient and spatially heterogeneous H_2_O_2_ levels in vivo.

Collectively, these results demonstrate that KPBIT@NPs integrate favorable structural stability with ROS-triggered release capability. Under non-oxidative conditions, the nanoparticles effectively retained ICT and TS IIA and minimized premature drug leakage, whereas oxidative stimulation induced phenylboronic ester cleavage, nanoparticle disassembly, and accelerated drug release. This stability–responsiveness balance provides a physicochemical basis for controlled drug delivery in oxidative stress-associated pathological microenvironments, including neurodegenerative diseases.

### 2.5 Cellular uptake, BBB translocation, and in vivo brain targeting of nanoparticles

**Fig. 2:**
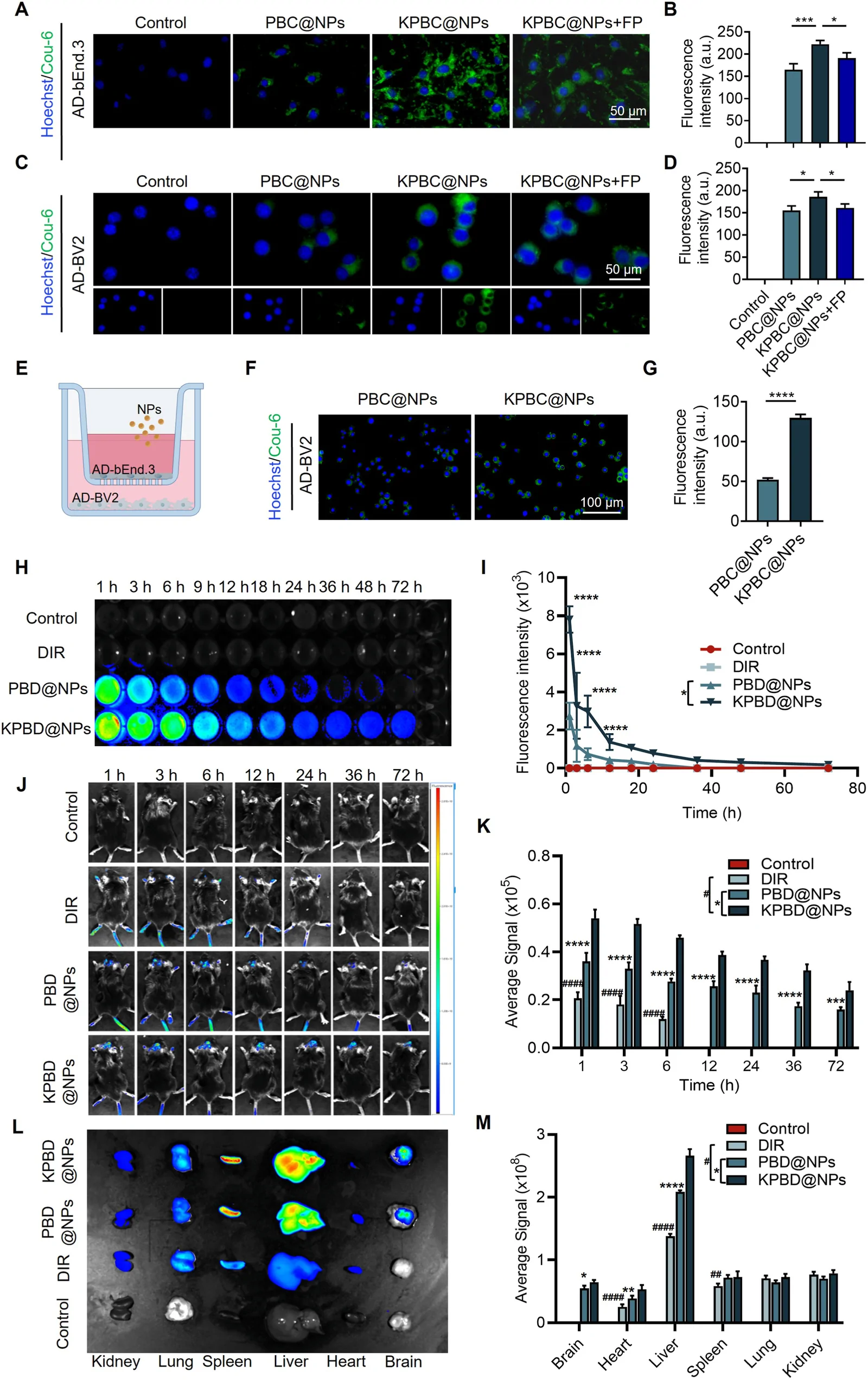
**Nanoformulations exhibit enhanced cellular uptake, BBB penetration, and brain targeting in vitro and in vivo**. (A) Representative fluorescence images of cellular uptake in AD-bEnd.3 cells treated with different formulations, Scale bar, 50 μm. (B) Semi-quantitative analysis of cellular uptake in (A) (n = 3). (C) Representative fluorescence images of cellular uptake in AD-BV2 cells, Scale bar, 50 μm. (D) Semi-quantitative analysis of cellular uptake in (C) (n = 3). (E) Schematic illustration of the Transwell-based in vitro BBB model. (F) Representative fluorescence images of uptake by AD-BV2 cells in the co-culture BBB model after treatment with different formulations, Scale bar, 100 μm. (G) Semi-quantitative analysis of AD-BV2 cellular uptake in (F) (n = 3). (H) Fluorescence signal intensity in rat blood at different time points after administration of different formulations. (I) Semi-quantitative analysis of blood fluorescence intensity in (H) (n = 3). (*: PBD@NPs vs KPBD@NPs) (J) Real-time in vivo fluorescence imaging of APP/PS1 mice after administration of different formulations. (K) Semi-quantitative analysis of fluorescence intensity in brain tissues corresponding to (J) (n = 3). (*: PBD@NPs vs KPBD@NPs; ^#^: DIR vs KPBD@NPs) (L) Ex vivo fluorescence imaging of major organs. (M) Semi-quantitative analysis of fluorescence intensity in tissues corresponding to (L) (n = 3) (*: PBD@NPs vs KPBD@NPs; ^#^: DIR vs KPBD@NPs). Data are presented as mean ± SD (*P < 0.05, **P < 0.01, ***P < 0.001, ****P < 0.0001, ^#^*P* < 0.05, ^##^*P* < 0.01, ^###^*P* < 0.001, ^####^*P* < 0.0001).

To evaluate the effect of KLV peptide modification on the cellular uptake of nanoformulations in an AD-related BBB model, bEnd.3 cells were co-stimulated with Aβ and lipopolysaccharide (LPS) to establish an in vitro BBB endothelial cell model under AD-like pathological conditions (AD-bEnd.3). Coumarin-6 (Cou-6) was used as a fluorescent probe to label non-modified nanoparticles (PBC@NPs) and KLV-modified nanoparticles (KPBC@NPs). Cellular uptake was examined using inverted fluorescence microscopy. As shown in Fig. 2A, blue fluorescence represents nuclear staining, whereas green fluorescence indicates the intracellular distribution of nanoparticles. Compared with the PBC@NPs group, KPBC@NPs exhibited significantly stronger green fluorescence intensity in bEnd.3 cells (***P < 0.001). The fluorescence was predominantly localized in the cytoplasm, indicating efficient cellular internalization. To further investigate the underlying mechanism, cells were pretreated with the receptor for advanced glycation end products (RAGE) inhibitor FPS-ZM1 (FP) before incubation with KPBC@NPs. FP pretreatment significantly reduced nanoparticle uptake, as evidenced by decreased intracellular fluorescence (*P < 0.05). The semi-quantitative analysis is shown in Fig. 2B. These results suggest that KLV peptide modification markedly enhances the interaction between nanoparticles and BBB endothelial cells under AD-like conditions. Given that RAGE is upregulated in AD and is involved in Aβ transport across the BBB, the Aβ-derived peptide sequence KLVFFAED may contribute to RAGE-associated recognition, thereby promoting the uptake of KPBC@NPs in pathological cell models [39]. In addition, flow cytometry analysis confirmed that FP pretreatment hindered nanoparticle uptake, consistent with the fluorescence imaging results; the corresponding data are shown in Supplementary Fig. S3A and S3C. Collectively, these findings indicate that peptide modification plays an important role in enhancing cellular uptake and provides a basis for subsequent BBB transport.

To further assess uptake in pathological immune cells, BV2 microglial cells were co-stimulated with Aβ and LPS to establish an in vitro model of overactivated microglia under AD-like conditions (AD-BV2). The cellular uptake of PBC@NPs, KPBC@NPs, and KPBC@NPs following FP pretreatment was then evaluated. As shown in Fig. 2C, KPBC@NPs exhibited significantly enhanced intracellular fluorescence in BV2 cells compared with PBC@NPs (*P < 0.05). This enhancement was attenuated after FP pretreatment, with a marked reduction in fluorescence intensity (*P < 0.05). Semi-quantitative results are shown in Fig. 2D. Furthermore, flow cytometry analysis also corroborated the fluorescence imaging findings; the corresponding data are shown in Supplementary Fig. S3B and S3D. These results further confirm that KLV peptide modification enhances nanoparticle uptake by pathologically activated microglia.

To systematically evaluate BBB translocation, an in vitro BBB co-culture model was established according to our previous study and a published protocol [[55], [56], [57]], with bEnd.3 cells seeded in the upper chamber and BV2 microglia cultured in the lower chamber. After 7 days of culture, the normal bEnd.3 monolayer reached a stable transendothelial electrical resistance (TEER) of 212.2 ± 12.6 Ω cm^2^, indicating the formation of a continuous endothelial monolayer with measurable barrier resistance. Following combined Aβ/LPS stimulation, the TEER value decreased to 189.1 ± 4.5 Ω cm^2^ (Supplementary Fig. S4A), suggesting that AD-like pathological stimulation partially impaired endothelial barrier integrity.

Because TEER values of bEnd.3 monolayers can vary depending on cell seeding density, culture duration, Transwell membrane properties, coating conditions, medium composition, and measurement systems, BBB model establishment was not evaluated using a single fixed TEER threshold. Instead, barrier formation and injury were assessed by integrating TEER measurement, sodium fluorescein (Na-FLU) permeability analysis, and immunofluorescence staining of tight-junction and cytoskeletal markers. After correction using cell-free Transwell inserts, the permeability coefficient of Na-FLU across the normal bEnd.3 monolayer was (1.92 ± 0.17) × 10^−6^ cm/s, whereas that of the AD-bEnd.3 model increased to (2.76 ± 0.32) × 10^−6^ cm/s. This increase indicated enhanced paracellular permeability after Aβ/LPS stimulation. Nevertheless, the Na-FLU permeability of the AD-bEnd.3 monolayer remained markedly lower than that of the cell-free Transwell control, which was (52.10 ± 0.57) × 10^−6^ cm/s (Supplementary Fig. S4B), demonstrating that the injured endothelial monolayer still retained substantial barrier-restrictive capacity rather than undergoing complete collapse.

Immunofluorescence staining was used as complementary structural evidence for the BBB model. Zonula occludens-1 (ZO-1), Occludin, and filamentous actin (F-actin) staining showed that bEnd.3 cells formed a relatively continuous and compact monolayer under normal culture conditions. Following Aβ/LPS stimulation, the junction-related staining appeared less continuous, and vascular endothelial growth factor (VEGF) expression showed an increasing trend, supporting a partially injured BBB-like phenotype rather than complete disruption of the endothelial monolayer (Supplementary Fig. S4C–J).

Taken together, the TEER, Na-FLU permeability, and immunofluorescence results verified the formation of a functional bEnd.3 endothelial barrier under normal culture conditions and supported the successful induction of an AD-like injured BBB phenotype after Aβ/LPS stimulation. Importantly, although the AD-like stimulation compromised barrier function, the AD-bEnd.3 monolayer still maintained measurable electrical resistance, an intact cellular monolayer, and significant restriction of small-molecule tracer diffusion. Therefore, this model represented a partially impaired but not completely disrupted BBB and was suitable for evaluating and comparing nanoparticle transendothelial transport under AD-like pathological conditions.

To assess BBB translocation and subsequent microglial uptake, AD-bEnd.3 cells were placed in the upper chamber, whereas AD-BV2 microglia were cultured in the lower chamber (Fig. 2E). As shown in Fig. 2F, KPBC@NPs produced stronger fluorescence signals in BV2 microglia in the lower chamber than PBC@NPs, indicating that KLV modification enhanced nanoparticle transport across the injured endothelial barrier and increased subsequent uptake by pathologically activated microglia. Semi-quantitative analysis further confirmed that KPBC@NPs achieved significantly higher BBB transport efficiency than PBC@NPs (****P < 0.0001), indicating that KLV modification contributes to improved endothelial barrier penetration (Fig. 2G). The enhanced BBB translocation may result from multiple synergistic factors. KLV peptide modification may improve interactions with Aβ-enriched pathological microenvironments or RAGE, thereby facilitating transport under disease-like conditions [58]. In addition, KPBC@NPs possess favorable physicochemical properties, including an appropriate particle size, low polydispersity, and a mildly negative surface charge. These characteristics may reduce nonspecific adsorption and enhance stability during transendothelial transport. Together, these properties may enable efficient BBB penetration and facilitate accumulation in pathological microglia.

To evaluate in vivo circulation and brain distribution, nanoparticles were labeled with the near-infrared probe DIR and intravenously injected into Sprague–Dawley (SD) rats. Blood samples were collected at different time points to measure fluorescence intensity. The circulation profiles of free DIR, non-targeted nanoparticles (PBD@NPs), and KLV-modified nanoparticles (KPBD@NPs) were compared. As shown in Fig. 2H, free DIR showed minimal blood fluorescence throughout the observation period, indicating rapid clearance. This rapid clearance may be associated with its high lipophilicity and poor aqueous solubility, which may promote nonspecific distribution and uptake by the reticuloendothelial system (RES). In contrast, both nanoparticle formulations showed significantly stronger and more sustained fluorescence signals, indicating prolonged circulation. This effect is likely attributable to the hydrophilic polysaccharide coating, which confers stealth-like properties and reduces RES uptake [59]. Notably, KPBD@NPs exhibited a longer circulation time than PBD@NPs, with detectable fluorescence even at 72 h post-injection. Semi-quantitative analysis is shown in Fig. 2I.

To further assess BBB penetration and brain distribution, in vivo real-time fluorescence imaging and ex vivo organ imaging were performed in AD mice. As shown in Fig. 2J and Supplementary Fig. S5, KPBD@NPs exhibited stronger brain accumulation signals at all measured time points, with sustained retention up to 72 h. Semi-quantitative analysis of brain fluorescence intensity further confirmed significantly higher signals in the KPBD@NPs group than in the PBD@NPs group (Fig. 2K). Ex vivo imaging of major organs at 72 h post-injection (Fig. 2L and M) revealed persistent and strong fluorescence signals in the brains of mice treated with KPBD@NPs, exceeding those observed in the non-targeted group. Together, our cellular uptake, in vitro BBB transport, and in vivo imaging results demonstrate that KLV-modified KPBC@NPs/KPBD@NPs exhibit enhanced pathological recognition, BBB-crossing ability, and brain accumulation, supporting their potential as brain-targeted nanocarriers for AD therapy. Nevertheless, we acknowledge that more direct intracerebral colocalization evidence was not obtained in the present study, which represents a technical limitation and an important aspect to be further addressed in our future work.

### 2.6 Evaluation of the synergistic neuroprotective effects of ICT and TS IIA and optimization of the co-loading ratio

**Fig. 3:**
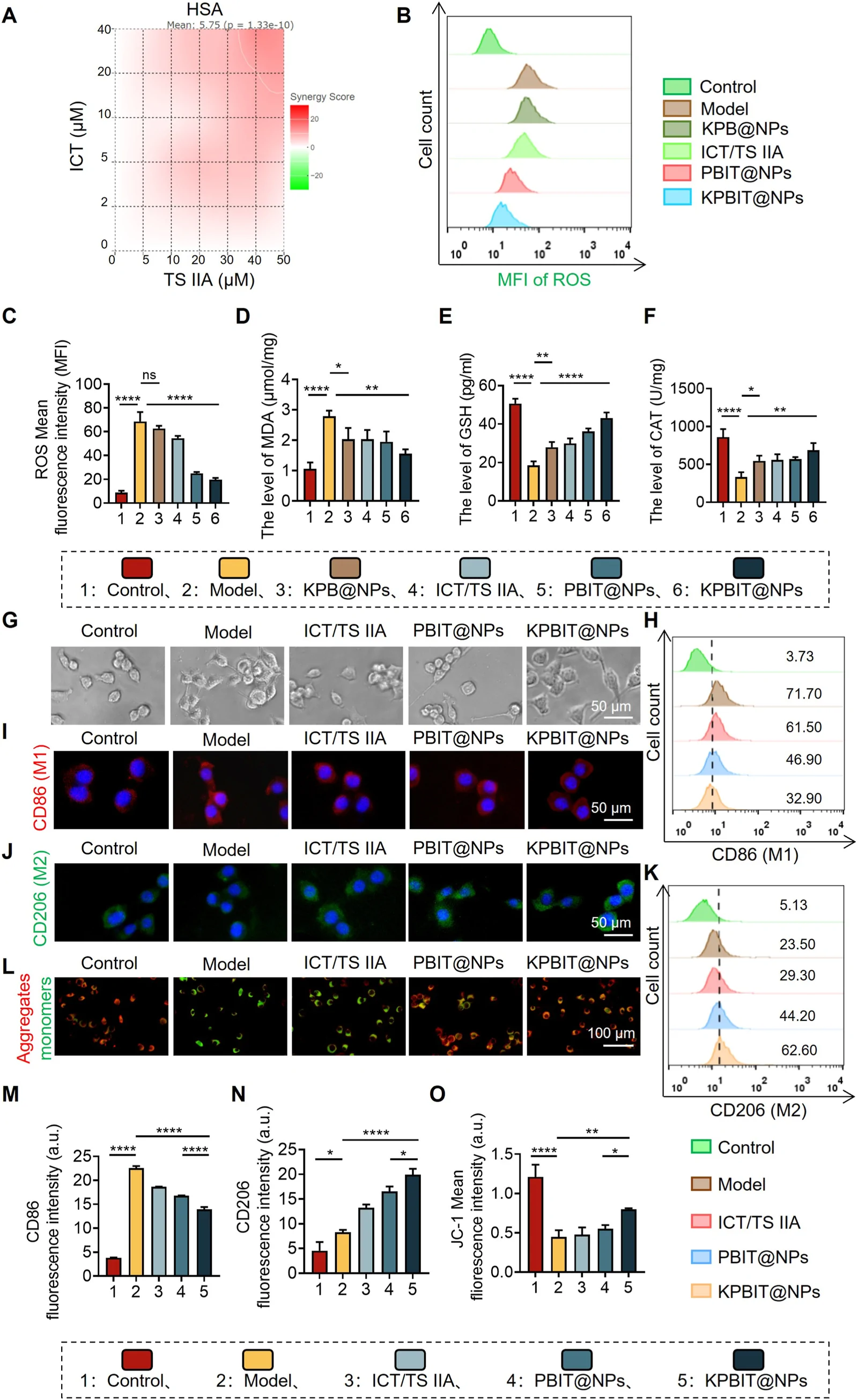
**Nanoformulations synergistically reduce oxidative stress, inhibit M1 polarization, and restore mitochondrial function in BV2 cells**. (A) Highest Single Agent (HSA) synergy analysis of free ICT and free TS IIA. (B) Flow cytometric analysis of intracellular ROS levels in BV2 cells treated with different formulations. (C) Quantification of ROS levels in (B). (D-F) Levels of oxidative stress-related biomarkers (MDA, GSH, and CAT) in the supernatant of BV2 cells after treatment. (G) Bright-field images of BV2 cells, Scale bar, 50 μm. (H) Flow cytometric analysis of CD86 expression. (I) Immunofluorescence images of CD86 expression (M1 marker) in BV2 cells, Scale bar, 50 μm. (J) Immunofluorescence images of CD206 expression (M2 marker) in BV2 cells, Scale bar, 50 μm. (K) Flow cytometric analysis of CD206 expression. (L) JC-1 staining showing mitochondrial membrane potential in BV2 cells, Scale bar, 100 μm. (M) Semi-quantitative analysis of CD86 expression in (I). (N) Semi-quantitative analysis of CD206 expression in (J). (O) Semi-quantitative analysis of mitochondrial membrane potential in (L). Data are presented as mean ± SD (n = 3, *P < 0.05, **P < 0.01, ***P < 0.001, ****P < 0.0001) (ns, not significant, *P* ≥ 0.05).

To systematically evaluate the synergistic neuroprotective effects of ICT and TS IIA in an AD-like microenvironment, BV2 microglial cells were co-stimulated with Aβ and LPS to establish an in vitro injury model. Cytoprotective efficacy was assessed primarily based on the rescue of cell viability across different treatment groups. On this basis, the synergistic effects of free ICT and TS IIA were quantitatively analyzed. Synergy was evaluated using the SynergyFinder platform, and a Synergy Score ≥ 10 was considered indicative of significant synergy. As shown in Fig. 3A, among the tested combinations, 3 M ratios—ICT:TS IIA = 2:5 (ratio 1), 4:5 (ratio 2), and 1:1 (ratio 3)—yielded Synergy Scores ≥ 10, indicating significant synergistic cytoprotective effects against Aβ + LPS-induced microglial injury within this ratio range. Based on the screening results of the free-drug combinations, nanoformulations were further prepared using the above molar ratios. These included nanoparticles loaded with ICT or TS IIA alone (KPBI@NPs and KPBT@NPs), as well as dual-drug co-loaded nanoparticles at the selected ratios (KPBIT@NPs-1, KPBIT@NPs-2, and KPBIT@NPs-3). The biological effects of single-drug and dual-drug nanoformulations were then systematically compared. Flow cytometry was used to assess BV2 microglial polarization, with particular attention to the shift from the pro-inflammatory M1 phenotype to the anti-inflammatory M2 phenotype. Compared with single-drug-loaded nanoparticles, dual-drug co-loaded formulations more effectively reduced the expression of the M1 marker CD86 and increased the expression of the M2 marker CD206. Among the tested ratios, KPBIT@NPs-3 (ICT:TS IIA = 1:1) showed the most pronounced inhibition of M1 polarization and promotion of M2 polarization (Supplementary Fig. S6A–D).

Chronically overactivated microglia have been shown to exacerbate neuroinflammation through the release of pro-inflammatory cytokines, promote neuronal apoptosis, and contribute to secondary brain injury [60]. Therefore, conditioned media from treated BV2 cells were collected and applied to HT22 neuronal cells to evaluate microglia-mediated indirect neuroprotective effects. Apoptosis analysis by flow cytometry showed that dual-drug-loaded nanoparticles significantly reduced HT22 neuronal apoptosis compared with single-drug formulations, with KPBIT@NPs-3 showing the most pronounced neuroprotective effect (Supplementary Fig. S7A–B). Consistently, Calcein-AM/PI dual staining combined with inverted fluorescence microscopy further confirmed enhanced HT22 cell viability in the KPBIT@NPs-3 group (Supplementary Fig. S8A–B). Collectively, these results demonstrate that the combination of ICT and TS IIA provides superior neuroprotection over monotherapy, as evidenced by synergistic screening, microglial phenotype reprogramming, and neuronal protection. Notably, the 1:1 M ratio showed the best overall performance across the functional assays. Therefore, this ratio was selected as the optimal co-loading ratio for subsequent in vitro and in vivo therapeutic and mechanistic studies.

### 2.7 KPBIT@NPs suppress oxidative stress and promote microglial phenotype reprogramming

Aβ deposition and damage-associated molecular patterns are recognized by pattern recognition receptors on microglia, thereby triggering polarization toward the pro-inflammatory M1 phenotype and inducing the release of cytokines such as interleukin-1β (IL-1β) and tumor necrosis factor-α (TNF-α). During inflammatory activation, excessive ROS are generated, leading to mitochondrial damage and further amplification of inflammatory signaling [61]. To assess the impact of different treatment strategies on oxidative stress, intracellular ROS levels in BV2 cells stimulated with Aβ + LPS were measured by flow cytometry. Experimental groups included the control, model, blank carrier (KPB@NPs), free drugs (ICT/TS IIA), non-targeted nanoparticles (PBIT@NPs), and targeted nanoparticles (KPBIT@NPs). As shown in Fig. 3B and C, all treatment groups reduced ROS levels to varying degrees compared with the model group, with KPBIT@NPs showing the most pronounced effect. To further evaluate the anti-inflammatory and antioxidant capacities of different treatments, the levels of interleukin-6 (IL-6), TNF-α, IL-1β, arginase-1 (Arg-1), and interleukin-10 (IL-10) were measured (Supplementary Fig. S9A–E). The blank carrier group exhibited moderate anti-inflammatory activity. In contrast, KPBIT@NPs significantly reduced the levels of the pro-inflammatory cytokines IL-6, TNF-α, and IL-1β, while markedly increasing the levels of the anti-inflammatory markers Arg-1 and IL-10. Concurrently, malondialdehyde (MDA) levels, glutathione (GSH) content, and catalase (CAT) activity were also assessed. As shown in Fig. 3D–F, both the blank carrier group and the free-drug group demonstrated moderate antioxidant activity. KPBIT@NPs, however, not only significantly decreased MDA levels but also markedly increased GSH content and CAT activity, showing the most pronounced antioxidant effects among the tested groups.

To systematically evaluate treatment-induced changes in BV2 cell morphology and inflammatory phenotype, bright-field imaging, morphometric analysis, immunofluorescence staining, and flow cytometry were performed following treatment with free ICT/TS IIA, PBIT@NPs, or KPBIT@NPs. As shown in Fig. 3G, BV2 cells in the control group were predominantly small and compact, with rounded or ovoid cell bodies and relatively few visible processes. In contrast, Aβ + LPS stimulation induced marked morphological heterogeneity, characterized by increased cell spreading, enlarged and flattened cell bodies, irregular cell contours, and local cell aggregation in the model group. Treatment with free ICT/TS IIA or PBIT@NPs partially altered these morphological changes, although both rounded and elongated cells remained visible. Notably, KPBIT@NPs treatment increased the occurrence of elongated, spindle-shaped, and process-bearing cells relative to the model group, while reducing the predominance of broadly spread and irregularly shaped cells. Quantitative analysis of cell circularity and process length was therefore performed to objectively characterize these treatment-associated morphological alterations (Supplementary Fig. S10A and S10B). These parameters were used only to assess changes in cell shape, spreading, and process extension and were not considered direct evidence of M1-or M2-like polarization.

The inflammatory phenotype of BV2 cells was independently evaluated using the pro-inflammatory-associated marker CD86 and the reparative-associated marker CD206. Immunofluorescence analysis showed that Aβ + LPS stimulation significantly upregulated both CD86 and CD206 expression, but CD86 was increased to a greater extent, shifting the CD86/CD206 balance toward a pro-inflammatory-like state in the model group (Fig. 3I–J and Fig. 3M–N). Treatment with free ICT/TS IIA, PBIT@NPs, and KPBIT@NPs decreased CD86 expression and further increased CD206 expression to varying degrees, with KPBIT@NPs producing the most pronounced effect. Flow cytometry analysis further confirmed these findings (Fig. 3H and K; Supplementary Fig. S10C and S10D). Aβ + LPS increased the proportions of both CD86-positive and CD206-positive BV2 cells; however, KPBIT@NPs more effectively reversed the pro-inflammatory shift by markedly reducing the CD86-positive population and further elevating the CD206-positive population compared with the free-drug combination or the non-targeted nanoparticle formulation. Collectively, these results indicate that KPBIT@NPs induce measurable remodeling of BV2 cell morphology and promote phenotypic reprogramming from a pro-inflammatory-like state toward a reparative-like state.

Mitochondrial dysfunction plays a central role in the vicious cycle linking inflammatory activation and oxidative stress. It not only amplifies pro-inflammatory signaling but also impairs cellular energy metabolism, thereby hindering the transition toward the anti-inflammatory phenotype [62]. Therefore, mitochondrial membrane potential in BV2 cells was assessed using the JC-1 probe. This parameter reflects mitochondrial membrane polarization and is widely used as an indicator of mitochondrial functional status; its decline indicates mitochondrial damage, reduced adenosine triphosphate (ATP) production, and cellular stress. As shown in Fig. 3L and O, mitochondrial membrane potential was significantly decreased in the model group, indicating mitochondrial dysfunction induced by inflammatory activation. Compared with free drugs and non-targeted nanoparticles, KPBIT@NPs significantly restored mitochondrial membrane potential, suggesting that the targeted nanoparticles not only suppress pro-inflammatory polarization but also preserve mitochondrial homeostasis and alleviate inflammation- and oxidative stress-induced damage. In summary, KPBIT@NPs exhibit distinct advantages by coordinately regulating microglial morphology, phenotype, and mitochondrial function. These effects were associated with enhanced M2 polarization, attenuated pro-inflammatory responses, reduced oxidative damage, and improved mitochondrial membrane potential. Together, these findings support the therapeutic potential of KPBIT@NPs in alleviating neuroinflammation and metabolic dysregulation in the AD microenvironment.

### 2.8 Network pharmacology and molecular docking suggest the synergistic mechanisms of ICT and TS IIA in AD

**Fig. 4:**
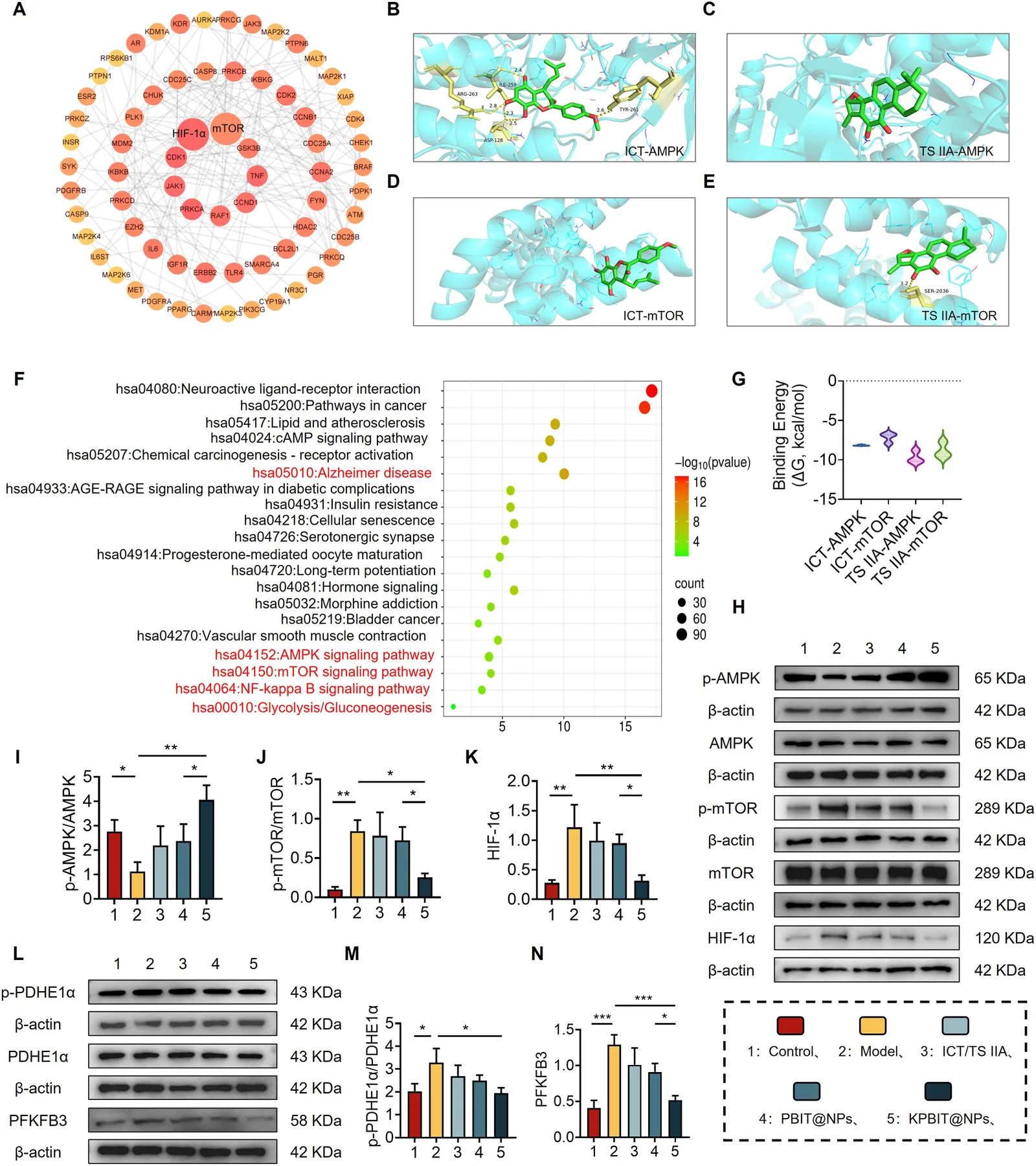
**ICT and TS IIA regulate BV2 polarization through activation of the AMPK-mTOR/HIF-1α signaling axis and metabolic reprogramming**. (A) Protein–protein interaction (PPI) network of core targets. (B–E) Molecular docking analysis showing binding interactions of ICT and TS IIA with AMPK and mTOR. (F) KEGG pathway enrichment analysis of potential therapeutic targets, highlighting the involvement of the AMPK and mTOR signaling pathways. (G) Quantitative analysis of binding energies corresponding to (B–E) (n = 3). (H) Western blot analysis of proteins in the AMPK and mTOR signaling pathway. (I–K) Quantification of p-AMPK/AMPK, p-mTOR/mTOR, and HIF-1α expression levels (n = 3). (L) Western blot analysis of metabolic enzymes PDHE1α and PFKFB3. (M − N) Quantification of p-PDHE1α/PDHE1α and PFKFB3 expression levels (n = 3). Data are presented as mean ± SD (*P < 0.05, **P < 0.01, ***P < 0.001, ****P < 0.0001).

Guided by the traditional therapeutic principle of “BuShen HuoXue”, which has been widely applied in cognitive decline-related disorders, *Epimedium*-derived ICT and TS IIA from *Salvia miltiorrhiza* were selected as representative active compounds. To systematically predict their potential synergistic anti-AD mechanisms, a network pharmacology approach was first employed to identify key targets and pathways. A total of 48 active compounds were collected from *Epimedium* and *Salvia miltiorrhiza*-related databases, and 736 putative drug targets were predicted accordingly. Meanwhile, 16,820 AD-related targets were retrieved from disease databases, including GeneCards and OMIM. Intersection analysis identified 688 common targets as potential therapeutic targets for further investigation (Supplementary Fig. S11A). A protein-protein interaction (PPI) network was then constructed using the STRING database and visualized in Cytoscape 3.9.1. Topological analysis identified key hub targets, including HIF-1α and mTOR, suggesting that these molecules may play important roles in mediating the therapeutic effects (Fig. 4A). To further clarify the relationships among drugs, compounds, targets, and disease, a “drug–compound–target–disease” network was constructed. The results showed that major active compounds of *Salvia miltiorrhiza* (e.g., TS IIA) and *Epimedium* (e.g., ICT and related glycosides) showed network associations with the identified core targets (Supplementary Fig. S11B). These findings suggest that ICT and TS IIA may exert anti-AD effects through multi-target synergistic regulation. To explore the biological functions and signaling pathways involved, Gene Ontology (GO) and Kyoto Encyclopedia of Genes and Genomes (KEGG) enrichment analyses were performed on the 688 intersecting targets. GO enrichment analysis indicated that these targets were mainly involved in protein phosphorylation and cellular responses to altered oxygen levels. The enriched cellular components included the cytoplasm, nucleoplasm, nucleus, and cytoskeleton, whereas the major molecular functions included protein binding, ATP binding, kinase activity, oxidoreductase activity, and transmembrane transporter activity (Supplementary Fig. S11C). KEGG pathway analysis identified 164 significantly enriched pathways (*P < 0.05). The top 20 enriched pathways (Fig. 4F) included the AMPK signaling pathway, mTOR signaling pathway, and neuroinflammation-related pathways. Together, these results offer a systems-level perspective on the potential mechanisms of the classical herb pair *Epimedium–Salvia miltiorrhiza* in AD, and suggest a possible convergence between traditional medicinal theory and key regulatory pathways such as AMPK, mTOR, and inflammation. To further evaluate whether ICT and TS IIA may directly interact with AMPK and mTOR, molecular docking simulations were performed against AMPK and mTOR as receptor proteins. Binding affinities (ΔG) were calculated using AutoDock Vina. ICT showed binding energies of (−8.17 ± 0.05) kcal/mol with AMPK and (−7.17 ± 0.52) kcal/mol with mTOR, whereas TS IIA exhibited binding energies of (−9.70 ± 0.64) kcal/mol with AMPK and (−8.83 ± 0.82) kcal/mol with mTOR (Fig. 4B–G). All binding energies were below −5.0 kcal/mol, suggesting potentially favorable binding interactions (Supplementary Table S5). Interaction analysis indicated that hydrogen bonding and hydrophobic interactions were the major forces stabilizing ligand–protein binding, supporting the possibility that ICT and TS IIA may interact with AMPK and mTOR. Taken together, network pharmacology and molecular docking collectively suggest that ICT and TS IIA may synergistically regulate AMPK/mTOR-related signaling in AD, with HIF-1α emerging as an additional hub target from network analysis. These findings may help explain how ICT and TS IIA contribute to the regulation of microglial immunometabolic balance in AD.

### 2.9 KPBIT@NPs reprogram the immunometabolic state of BV2 microglia via activation of AMPK and inhibition of the mTOR/HIF-1α axis

In the chronic inflammatory microenvironment of AD, microglia undergo metabolic reprogramming characterized by impaired energy sensing and a shift from mitochondrial OXPHOS to glycolysis, which favors a pro-inflammatory phenotype. This metabolic remodeling is closely associated with inflammatory status and phenotypic polarization [63,64]. AMPK is a central energy-sensing kinase, and its activity, reflected by the p-AMPK/AMPK ratio, is essential for maintaining metabolic homeostasis. In contrast, the mTOR/HIF-1α signaling axis promotes the expression of glycolytic genes and drives pro-inflammatory metabolic programs. Because AMPK and mTOR are reciprocally regulated, disruption of the AMPK-mTOR/HIF-1α network represents an important molecular basis for inflammatory metabolic dysregulation in microglia [65,66]. To determine whether KPBIT@NPs mediate metabolic reprogramming through this regulatory network, the p-AMPK/AMPK ratio, p-mTOR/mTOR ratio, and HIF-1α expression were evaluated in BV2 cells. As shown in Fig. 4H–K, the model group exhibited a significant decrease in the p-AMPK/AMPK ratio compared with the control group, suggesting impaired AMPK signaling and disrupted energy-sensing homeostasis under inflammatory and toxic stimulation. Consistently, the p-mTOR/mTOR ratio and HIF-1α expression were significantly increased in the model group. mTOR hyperactivation is closely associated with the glycolytic shift in pro-inflammatory microglia, whereas HIF-1α directly upregulates glycolytic genes such as 6-phosphofructo-2-kinase/fructose-2,6-bisphosphatase 3 (PFKFB3), thereby further enhancing glycolytic flux. These findings suggest that inflammatory and toxic stimuli activate the mTOR/HIF-1α axis while weakening AMPK-mediated negative regulation. In contrast, treatment with KPBIT@NPs significantly increased the p-AMPK/AMPK ratio, indicating restoration of cellular energy-sensing capacity (Fig. 4H and I). Reactivation of AMPK contributes to suppression of mTOR hyperphosphorylation and reduces the drive toward glycolytic metabolism, thereby promoting recovery of oxidative metabolic homeostasis. Concurrently, KPBIT@NPs markedly decreased the p-mTOR/mTOR ratio and HIF-1α expression (Fig. 4H, J and 4K), demonstrating effective inhibition of this pro-inflammatory metabolic axis. This coordinated regulation of AMPK and mTOR/HIF-1α provides a mechanistic basis for the subsequent metabolic and functional improvements. Importantly, compared with free ICT/TS IIA and non-targeted PBIT@NPs, KPBIT@NPs exhibited more pronounced regulatory effects on this signaling axis. These results suggest that co-delivery of ICT and TS IIA within a targeted nanocarrier enhances regulation of the AMPK-mTOR/HIF-1α signaling axis.

To further elucidate the effects of KPBIT@NPs on inflammatory metabolism, key nodes involved in glycolysis and mitochondrial oxidative metabolism were examined (Fig. 4L–N). In the model group, PFKFB3 expression was markedly upregulated. Because PFKFB3 controls a rate-limiting step in glycolysis, its elevation provides molecular evidence of enhanced glycolytic flux. Meanwhile, the inhibitory phosphorylation level of pyruvate dehydrogenase E1 subunit alpha (PDHE1α) was significantly increased, indicating restricted pyruvate entry into the mitochondrial tricarboxylic acid cycle and a preferential shift toward glycolysis. Treatment with KPBIT@NPs significantly reduced PFKFB3 expression and decreased p-PDHE1α levels. These findings suggest that KPBIT@NPs not only suppress a key glycolytic regulator but also relieve inhibition of the pyruvate dehydrogenase (PDH) complex, thereby restoring PDH activity and promoting pyruvate flux into the tricarboxylic acid cycle, suggesting partial restoration of mitochondrial OXPHOS capacity.

**Fig. 5:**
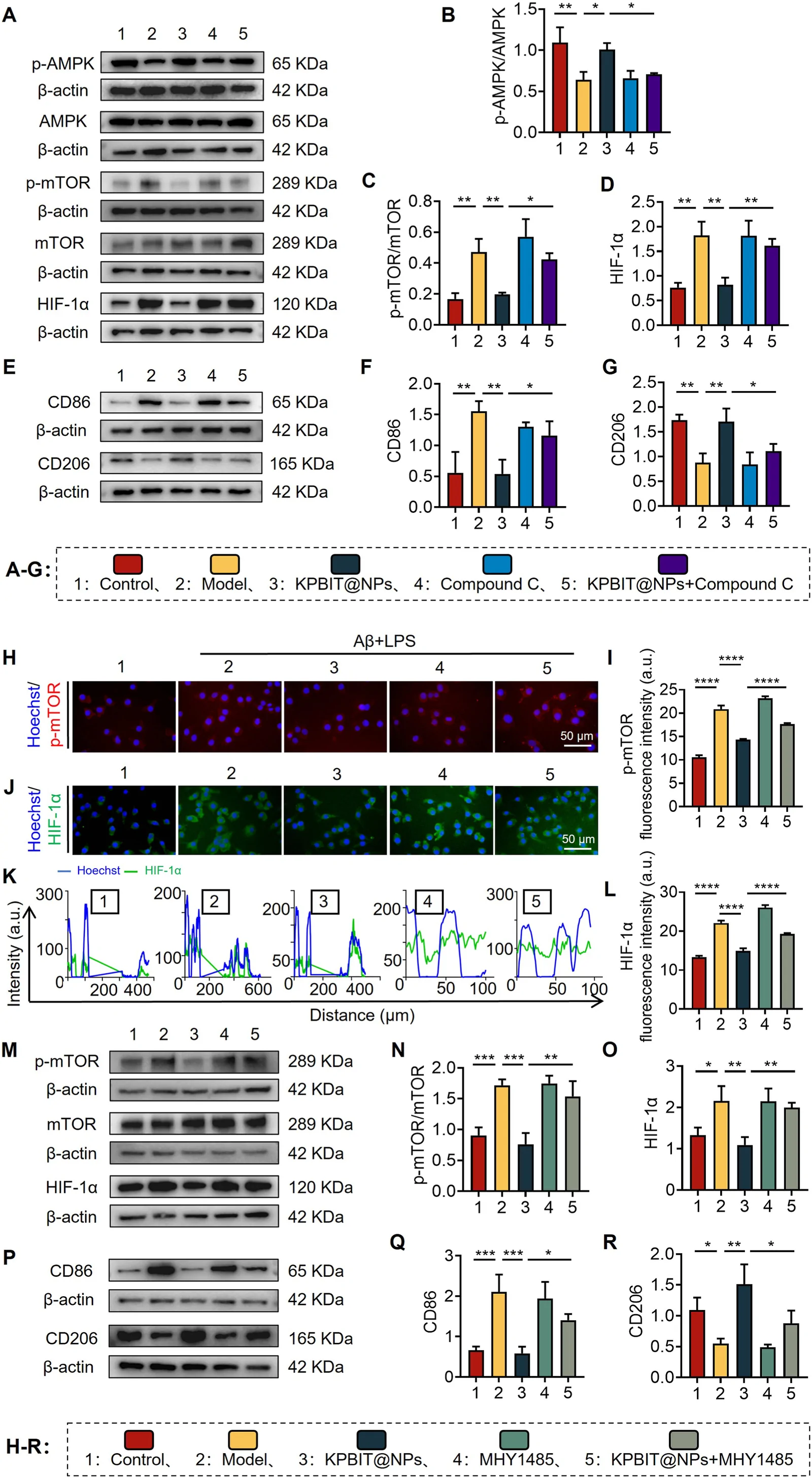
**Pharmacological validation of AMPK-mTOR/HIF-1α signaling in regulating BV2 polarization by nanoformulations.** (A) Western blot analysis of proteins in the AMPK–mTOR signaling pathway across different treatment groups. (B–D) Quantification of p-AMPK/AMPK, p-mTOR/mTOR, and HIF-1α expression levels corresponding to (A). (E) Western blot analysis of CD86 (M1 marker) and CD206 (M2 marker). (F–G) Quantification of CD86 and CD206 expression corresponding to (E). (H) Immunofluorescence images of p-mTOR expression in BV2 cells, Scale bar, 50 μm. (I) Semi-quantitative analysis of p-mTOR fluorescence intensity in (H). (J) Immunofluorescence images of HIF-1α expression, Scale bar, 50 μm. (K) Fluorescence intensity profiles of HIF-1α and nuclear staining, showing altered nuclear localization. (L) Semi-quantitative analysis of HIF-1α fluorescence intensity in (J). (M) Western blot analysis of p-mTOR, total mTOR, HIF-1α, and the loading control under pharmacological modulation. (N–O) Quantification of p-mTOR/mTOR and HIF-1α expression corresponding to (M). (P) Western blot analysis of CD86 and CD206 expression under pathway intervention. (Q–R) Quantification of CD86 and CD206 expression corresponding to (P). Groups (A–G): 1. Control, 2. Model, 3. KPBIT@NPs, 4. Compound C, 5. KPBIT@NPs + Compound C. Groups (H–R): 1. Control, 2. Model, 3. KPBIT@NPs, 4. MHY1485, 5. KPBIT@NPs + MHY1485. Data are presented as mean ± SD (n = 3, *P < 0.05, **P < 0.01, ***P < 0.001, ****P < 0.0001).

To further assess whether AMPK and mTOR are required for KPBIT@NPs-mediated regulation of microglial immunometabolism, pharmacological interventions were performed using the AMPK inhibitor Compound C and the mTOR activator MHY1485. As shown in Fig. 5, pretreatment with Compound C significantly attenuated the ability of KPBIT@NPs to increase the p-AMPK/AMPK ratio (*P < 0.05) and weakened their inhibitory effects on p-mTOR and HIF-1α (Fig. 5A–D). In parallel, KPBIT@NPs-induced downregulation of the M1 marker CD86 and upregulation of the M2 marker CD206 were markedly diminished (Fig. 5E–G). To further assess the role of mTOR activation, BV2 cells were pretreated with MHY1485, followed by immunofluorescence analysis of p-mTOR expression and its downstream effects. Representative images (Fig. 5H) showed that KPBIT@NP treatment markedly reduced p-mTOR fluorescence intensity compared with the model group, indicating effective suppression of aberrant mTOR activation. This inhibitory effect was significantly reversed by MHY1485 treatment, as evidenced by a strong increase in p-mTOR fluorescence. Quantitative analysis (Fig. 5I) further confirmed that the p-mTOR signal in the KPBIT@NPs + MHY1485 group was significantly higher than that in the KPBIT@NPs group alone (****P < 0.0001), suggesting that mTOR activation partially counteracts the inhibitory effect of KPBIT@NPs. Consistent with the p-mTOR expression pattern, immunofluorescence images (Fig. 5J) and quantitative analysis (Fig. 5L) of HIF-1α revealed that KPBIT@NPs significantly reduced HIF-1α fluorescence intensity, whereas MHY1485 co-treatment restored its expression (****P < 0.0001). Because HIF-1α activation is associated with nuclear translocation, its subcellular localization was further analyzed (Fig. 5K). Co-localization analysis of nuclear staining (blue) and HIF-1α fluorescence (green) demonstrated reduced nuclear localization in the KPBIT@NPs-treated group compared with the model group. This effect was partially reversed in the presence of MHY1485, further supporting the regulatory role of mTOR signaling in HIF-1α activation and subcellular localization.

Western blot analysis further validated these findings at the protein level. As shown in Fig. 5M − O, activation of mTOR by MHY1485 significantly attenuated the inhibitory effects of KPBIT@NPs on p-mTOR and HIF-1α, confirming that mTOR activation can partially offset KPBIT@NP-mediated pathway suppression. In addition, modulation of this pathway influenced microglial polarization. KPBIT@NPs increased the expression of the anti-inflammatory marker CD206 and decreased the pro-inflammatory marker CD86. However, these effects were partially reversed upon co-treatment with MHY1485 (F ig. 5P–R), indicating that mTOR activation compromises the immunoregulatory effects of KPBIT@NPs.

These findings indicate that AMPK activation together with mTOR inhibition represents a key mechanism by which KPBIT@NPs drive phenotypic transition and metabolic reprogramming. AMPK is a central energy-sensing kinase whose activation suppresses mTOR activity and thereby favors oxidative metabolism over glycolysis. We next evaluated the regulatory effects of KPBIT@NPs on key metabolic indicators to determine how pathway modulation reshapes cellular energy dynamics.

**Fig. 6:**
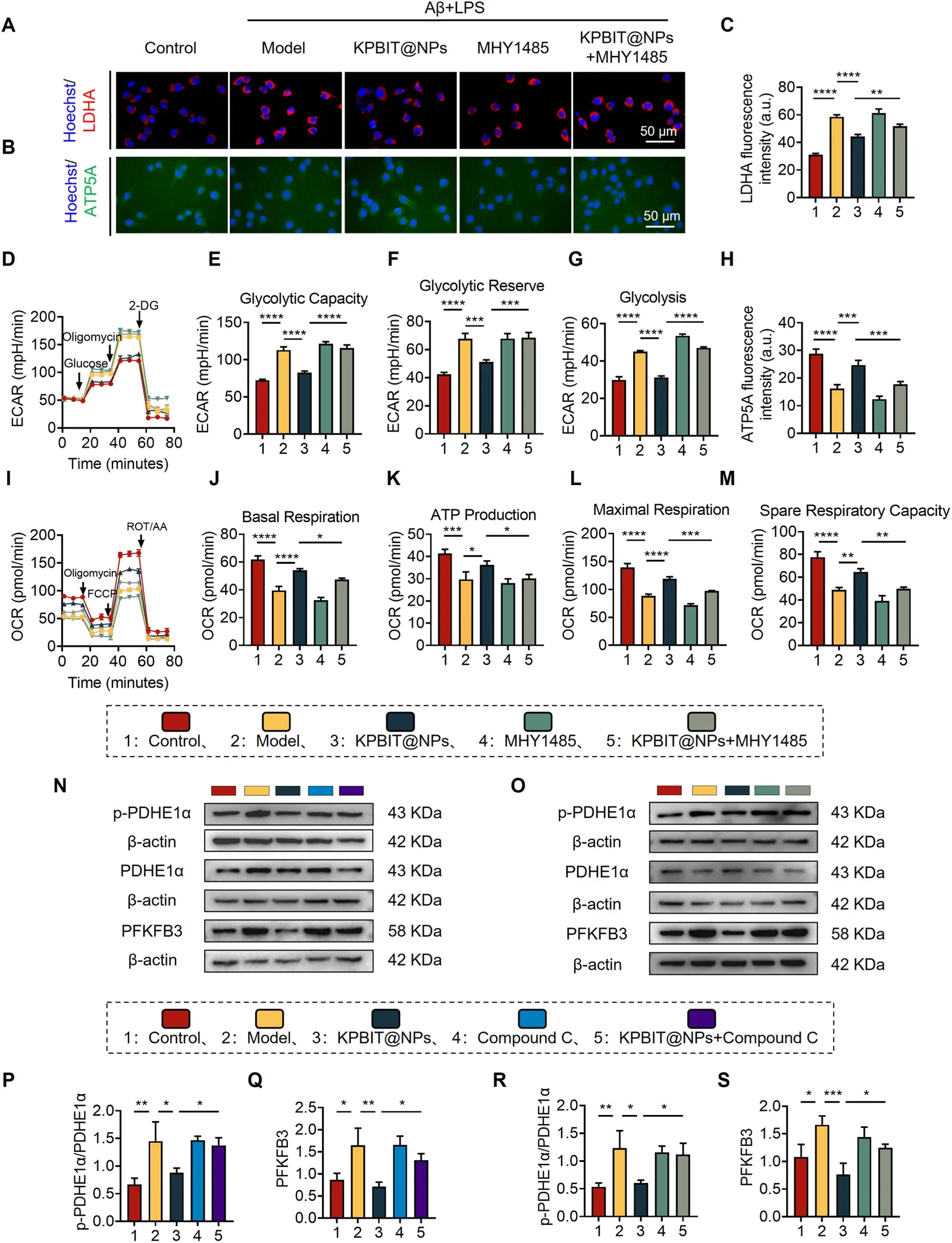
**Nanoformulations reprogram BV2 cell metabolism from glycolysis to oxidative phosphorylation via the AMPK–mTOR signalin**g pathway. (A) Immunofluorescence images of LDHA (glycolytic marker) expression in BV2 cells, Scale bar, 50 μm. (B) Immunofluorescence images of ATP5A (mitochondrial oxidative phosphorylation marker) expression, Scale bar, 50 μm. (C) Semi-quantitative analysis of LDHA fluorescence intensity corresponding to (A). (D) Representative ECAR curves. (E–G) Quantification of glycolytic capacity, glycolytic reserve, and glycolytic activity, indicating suppression of glycolysis. (H) Semi-quantitative analysis of ATP5A fluorescence intensity corresponding to (B). (I) Representative OCR curves. (J–M) Quantification of basal respiration, ATP production, maximal respiration, and spare respiratory capacity, indicating enhanced mitochondrial oxidative phosphorylation. (N) Western blot analysis of metabolic enzymes (PDHE1α and PFKFB3) under AMPK inhibition (Compound C). (O) Western blot analysis of metabolic enzymes under mTOR activation (MHY1485). (P–Q) Quantification of protein expression corresponding to (N). (R–S) Quantification of protein expression corresponding to (O). Groups (N): 1. Control, 2. Model, 3. KPBIT@NPs, 4. Compound C, 5. KPBIT@NPs + Compound C. Groups (O): 1. Control, 2. Model, 3. KPBIT@NPs, 4. MHY1485, 5. KPBIT@NPs + MHY1485. Data are presented as mean ± SD (n = 3, *P < 0.05, **P < 0.01, ***P < 0.001, ****P < 0.0001).

The expression patterns of lactate dehydrogenase A (LDHA), a glycolytic marker, and ATP5A, a marker of mitochondrial OXPHOS, reflect metabolic preference under inflammatory conditions. LDHA catalyzes the conversion of pyruvate to lactate and serves as a hallmark of glycolytic reprogramming, whereas ATP5A is a core subunit of the mitochondrial ATP synthase complex and is associated with OXPHOS capacity and cellular energy output [67,68]. Immunofluorescence analysis (Fig. 6A–C and Fig. 6H) showed that KPBIT@NPs markedly reduced LDHA fluorescence intensity compared with the model group, indicating effective suppression of inflammation-induced glycolysis. Upon pretreatment with MHY1485, the inhibitory effect of KPBIT@NPs on LDHA was significantly attenuated, and fluorescence intensity differed significantly from that in the KPBIT@NPs-only group. This finding suggests that mTOR activation partially reverses the glycolysis-suppressive effect of KPBIT@NPs. In parallel, ATP5A fluorescence intensity was markedly increased in the KPBIT@NPs-treated group, indicating restoration of mitochondrial OXPHOS. This effect was significantly weakened after co-treatment with MHY1485, further indicating that mTOR activation impairs the ability of KPBIT@NPs to restore oxidative metabolism.

To functionally validate dynamic metabolic changes, real-time metabolic flux analysis was performed to measure the oxygen consumption rate (OCR) and extracellular acidification rate (ECAR). Compared with the control group, the model group exhibited significantly elevated ECAR and reduced OCR, indicating a metabolic shift from mitochondrial OXPHOS toward glycolysis under inflammatory conditions. In contrast, KPBIT@NPs treatment significantly reduced glycolytic capacity, glycolytic reserve, and overall glycolytic activity as reflected by ECAR parameters (Fig. 6D–G). Meanwhile, basal respiration, ATP production, maximal respiration, and spare respiratory capacity were all significantly increased in the KPBIT@NPs group (Fig. 6I–M), indicating substantial recovery of OXPHOS.

Western blot analysis further examined PDH activity and key glycolytic enzymes (Fig. 6N and 6P–Q). PDH activity is regulated by the phosphorylation state of its E1α subunit, and a lower p-PDHE1α/PDHE1α ratio indicates higher PDH activity and a metabolic shift toward mitochondrial OXPHOS. In contrast, increased expression of the rate-limiting enzyme PFKFB3 is a hallmark of enhanced glycolysis. Compared with the model group, KPBIT@NPs significantly decreased the p-PDHE1α/PDHE1α ratio and PFKFB3/β-actin expression, confirming suppression of glycolysis and promotion of oxidative metabolism at the molecular level. However, co-treatment with the AMPK inhibitor Compound C markedly attenuated the regulatory effects of KPBIT@NPs on both p-PDHE1α/PDHE1α and PFKFB3 (Fig. 6N and 6P–Q), indicating that AMPK activation is required for KPBIT@NPs-mediated metabolic reprogramming. Similarly, co-treatment with the mTOR activator MHY1485 significantly impaired the modulation of these molecular markers by KPBIT@NPs (Fig. 6O and 6R–S), further demonstrating that inhibition of mTOR signaling is essential for the metabolic shift induced by KPBIT@NPs. In summary, in vitro experiments in BV2 microglia demonstrate that KPBIT@NPs activate AMPK while inhibiting the mTOR/HIF-1α signaling axis. This dual regulation suppresses inflammation-associated glycolysis, restores mitochondrial OXPHOS, and promotes the transition of microglia from the pro-inflammatory M1 phenotype to the anti-inflammatory M2 phenotype. This coordinated regulation of metabolism and phenotype provides a cellular basis for the subsequent neuroprotective effects observed in vivo.

### 2.10 Indirect neuroprotective effects of KPBIT@NPs via modulation of microglial paracrine signaling

**Fig. 7:**
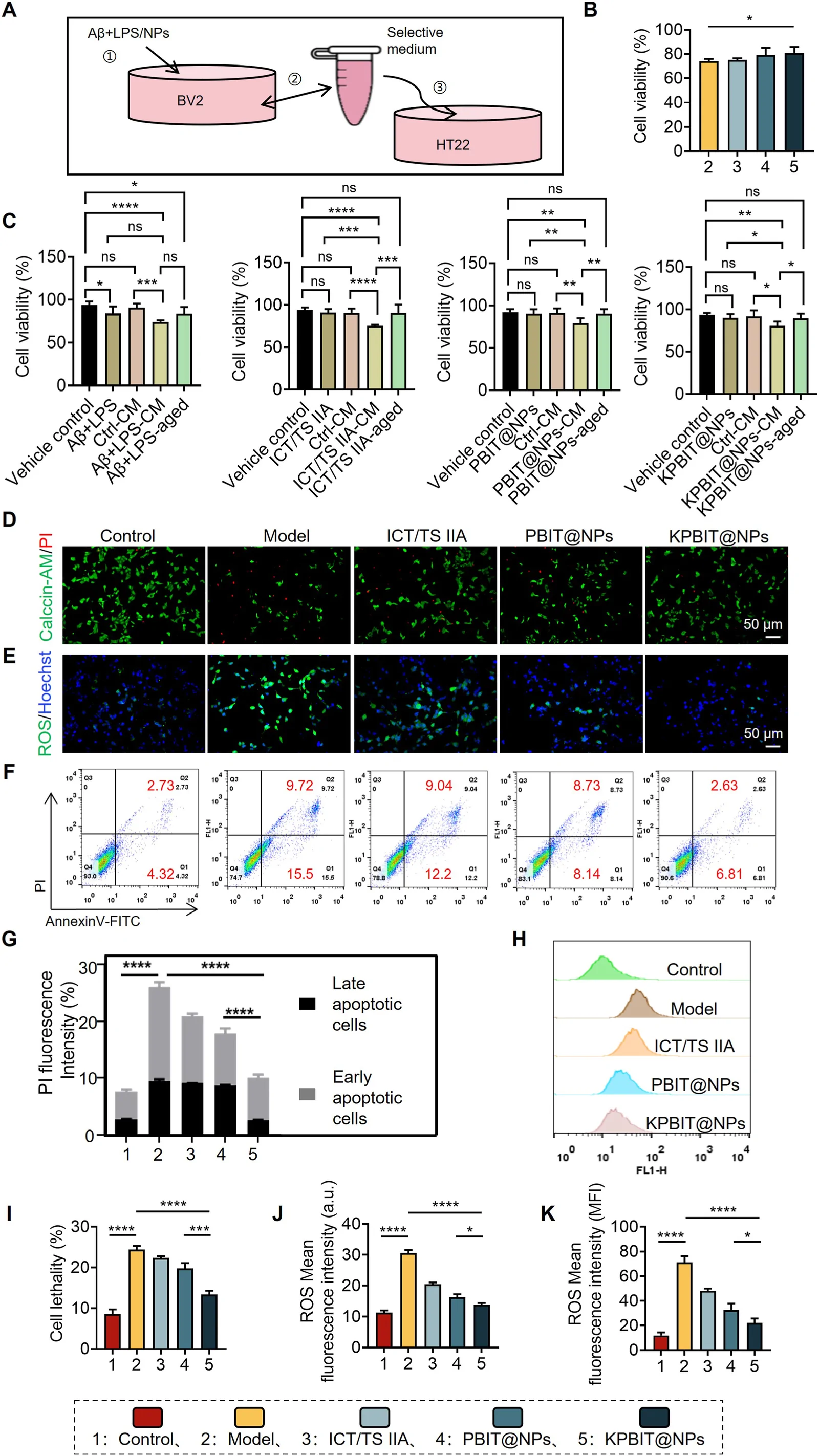
**Microglial reprogramming-mediated neuroprotection against neuronal oxidative stress and** apoptosis. (A) Schematic illustration of HT22 neurons cultured with CM derived from BV2 cells. (B) Cell viability of HT22 cells treated with CM from different groups (n = 6). (C) Control experiments to exclude direct effects of residual stimuli (Aβ + LPS, ICT/TS IIA, PBIT@NPs, KPBIT@NPs) on HT22 cells (n = 6). (D) Representative live/dead staining images of HT22 cells after 12 h incubation with CM, Scale bar, 50 μm. (E) Representative fluorescence images of intracellular ROS levels in HT22 cells, Scale bar, 50 μm. (F) Flow cytometry analysis of apoptosis in HT22 cells. (G) Quantification of apoptosis rate corresponding to (F) (n = 3). (H) Flow cytometry analysis of ROS levels in HT22 cells. (I) Quantification of cell death rate corresponding to (D) (n = 3). (J) Semi-quantitative analysis of ROS fluorescence intensity corresponding to (E) (n = 3). (K) Quantification of ROS levels corresponding to (H) (n = 3). Data are presented as mean ± SD (*P < 0.05, **P < 0.01, ***P < 0.001, ****P < 0.0001) (ns, not significant, *P* ≥ 0.05).

To evaluate the indirect regulatory effects of conditioned medium (CM) derived from BV2 microglia under different treatments on neuronal cells, a standardized workflow for BV2 CM collection was first established, as illustrated in Fig. 7A. To clarify whether the observed neurotoxicity was mediated by BV2-derived CM rather than by residual stimulants in the culture system, multiple control conditions were systematically included. These controls comprised fresh complete medium (vehicle control), fresh medium containing stimulants, CM from untreated BV2 cells (Ctrl-CM), CM from stimulant-treated BV2 cells, and stimulant-containing medium incubated for the same duration as the treatment groups without cell exposure. As shown in Fig. 7C, among these control groups, only CM derived from stimulant-treated BV2 cells induced a significant decrease in HT22 cell viability, whereas all other groups (fresh medium containing stimulants, Ctrl-CM, and stimulant-containing medium incubated without cells) showed no significant difference compared with the vehicle control. These results largely excluded the possibility that residual stimulants in the CM directly caused toxicity to HT22 cells, indicating that the observed neurotoxicity was primarily mediated by BV2-derived CM.

Following this validation, the indirect neuroprotective effects of KPBIT@NPs via modulation of microglial polarization were further investigated. HT22 neuronal cells were treated with BV2-derived CM under different conditions, and cell viability, oxidative stress, and related parameters were assessed. As shown in Fig. 7B, compared with the model group, CM derived from KPBIT@NPs-treated BV2 cells significantly improved HT22 cell viability, indicating a pronounced neuroprotective effect. Calcein-AM/PI live/dead staining further confirmed these findings (Fig. 7D), with increased green fluorescence (live cells) and reduced red fluorescence (dead cells) in the KPBIT@NPs group. Quantitative analysis (Fig. 7I) demonstrated that the cell death rate in the KPBIT@NPs group was significantly lower than that in the model group and showed superior protection compared with PBIT@NPs. Flow cytometry was subsequently employed to quantify apoptosis in HT22 cells. The results showed a marked increase in apoptosis under model conditions, whereas CM from KPBIT@NPs-treated BV2 cells significantly reduced the proportion of apoptotic cells (Fig. 7F). Quantitative analysis (Fig. 7G) was highly consistent with the viability and cell death data described above. Notably, CM from KPBIT@NPs-treated BV2 cells also significantly reduced early-stage apoptosis in neuronal cells. These findings indicate that CM derived from KPBIT@NPs-treated BV2 cells not only reduced overall neuronal cell death but also effectively inhibited the initiation of apoptotic pathways. Given that oxidative stress is a key pathological factor in neuronal injury, intracellular ROS levels in HT22 cells were further assessed. Representative fluorescence images (Fig. 7E) showed that CM from KPBIT@NPs-treated BV2 cells markedly reduced ROS accumulation in HT22 cells, as confirmed by quantitative analysis (Fig. 7J). Flow cytometry was then performed to further quantify ROS levels. The flow cytometric histograms are presented in Fig. 7H, with corresponding quantitative results in Fig. 7K. These findings were consistent with the imaging results and further demonstrated that KPBIT@NP-modulated BV2 CM significantly alleviated oxidative damage and neuronal cell death. Collectively, these results demonstrate that BV2-derived CM modulated by KPBIT@NPs significantly attenuates oxidative stress and cell death in HT22 neurons, thereby exerting indirect neuroprotective effects. These findings further support the notion that KPBIT@NPs mitigate secondary neuronal injury by reshaping microglial functional states and provide important cellular evidence for their in vivo neuroprotective potential.

### 2.11 KPBIT@NPs improve cognitive function and alleviate neuropathological damage in an AD mouse model

**Fig. 8:**
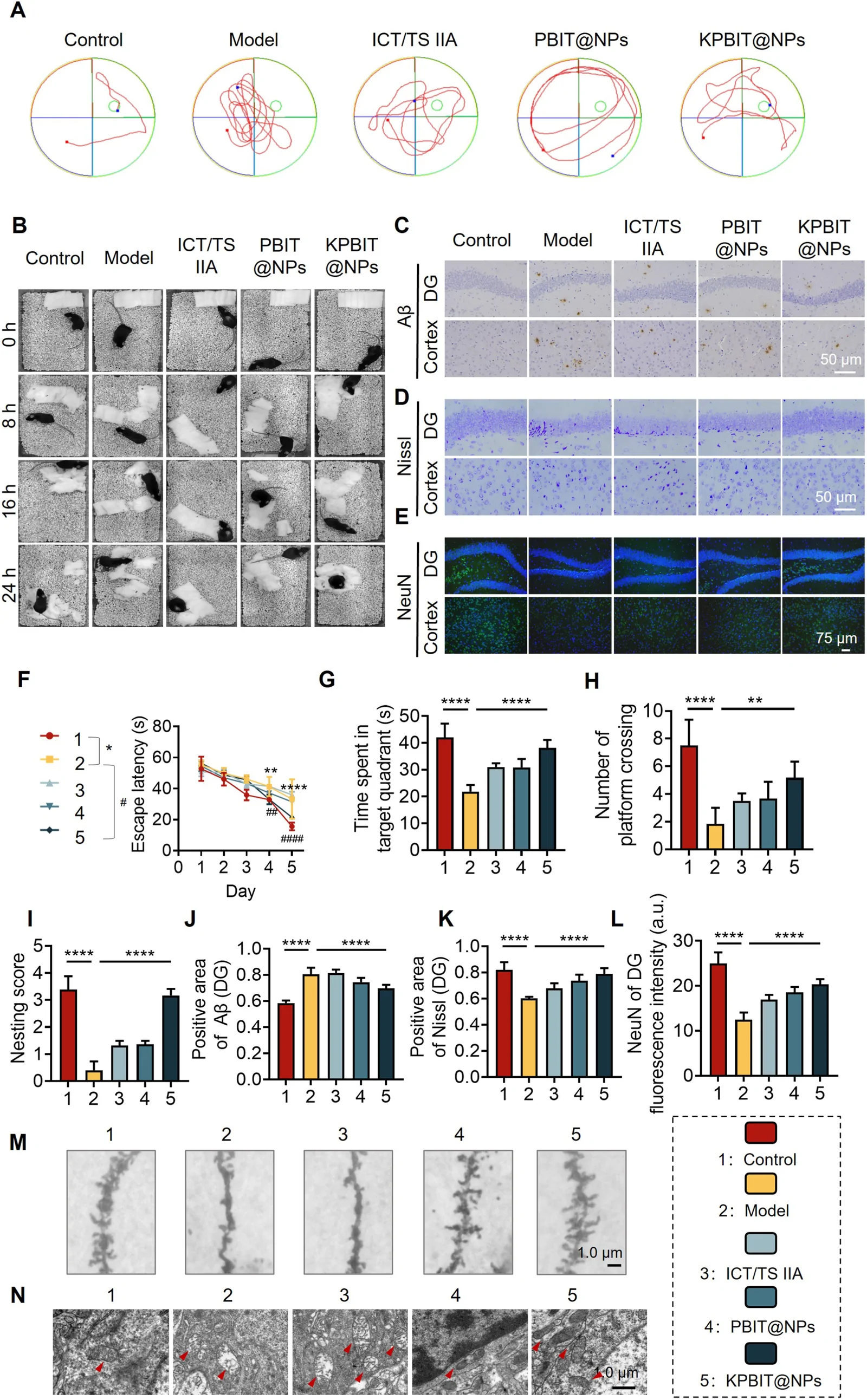
**Nanoformulations alleviate cognitive impairment, Aβ pathology, and neuronal damage in APP/PS1 mice**. (A) Representative swimming trajectories of mice in the Morris water maze on day 6. (B) Representative images of nesting behavior within 24 h. (C) Immunohistochemical staining of Aβ plaques in the cortex and hippocampal DG, Scale bar, 50 μm. (D) Nissl staining of neuronal structure in the cortex and hippocampal DG region, Scale bar, 50 μm. (E) Immunofluorescence staining of NeuN (neuronal marker), Scale bar, 75 μm. (F) Escape latency. (*: Control vs Model; ^#^: KPBIT@NPs vs Model). (G) Time spent in the target quadrant. (H) Number of platform crossings. (I) Quantification of nesting scores. (J) Semi-quantitative analysis of Aβ plaque burden. (K) Semi-quantitative analysis of the positive area of Nissl bodies in the hippocampal DG. (L) Semi-quantitative analysis of NeuN fluorescence intensity. (M) Golgi staining showing dendritic morphology in hippocampal neurons, Scale bar, 1.0 μm. (N) Ultrastructural analysis of mitochondrial morphology, red arrows indicate mitochondria, Scale bar, 1.0 μm. Data are presented as mean ± SD (n = 6, *P < 0.05, **P < 0.01, ***P < 0.001, ****P < 0.0001). (For interpretation of the references to colour in this figure legend, the reader is referred to the Web version of this article.)

Based on the in vitro pharmacological results, we further evaluated the in vivo therapeutic efficacy of the targeted formulation KPBIT@NPs in an AD mouse model. Cognitive performance, daily behavioral function, and brain pathology were systematically assessed. In the Morris water maze test, mice in the model group exhibited dispersed swimming trajectories during probe trials and lacked a clear preference for the target quadrant, indicating significant impairment in spatial learning and memory. In contrast, KPBIT@NPs-treated mice displayed search trajectories more concentrated in the target quadrant (Fig. 8A). Quantitative analysis further revealed that KPBIT@NPs significantly shortened escape latency, increased time spent in the target quadrant, and enhanced platform crossing frequency compared with the model and other treatment groups (Fig. 8F–H), indicating its efficacy in ameliorating AD-related spatial cognitive deficits. Nest-building behavior, used to evaluate daily functional capacity, was severely impaired in model mice, as reflected by loosely structured nests. Following KPBIT@NPs treatment, mice constructed nests with more intact morphology and compact structure (Fig. 8B). Quantitative nest scores were significantly higher in the KPBIT@NPs group than in the model group (Fig. 8I), indicating that this formulation significantly improves AD-associated impairments in daily activities.

To determine whether behavioral improvements were accompanied by mitigation of Aβ pathology, brain sections were subjected to Aβ immunohistochemistry. Extensive and densely stained Aβ plaques were observed in the hippocampus and cortex of model mice. In contrast, KPBIT@NPs treatment significantly reduced both plaque area and staining intensity (Fig. 8C), with quantitative analysis confirming statistical significance (Fig. 8J; Supplementary Fig. S13A), providing histopathological evidence supporting the cognitive protective effects of KPBIT@NPs. To further validate the neuroprotective effects in vivo, multiple morphological analyses were performed on brain tissues, including H&E staining, Nissl staining, neuronal nuclei (NeuN) immunofluorescence, Golgi staining, and TEM for mitochondrial ultrastructure. H&E staining revealed overall tissue structure and pathological damage. Compared with the blank control, model mice exhibited pronounced tissue disorganization and neuronal abnormalities. Treatment with various formulations improved structural damage to varying degrees, with KPBIT@NPs-treated mice showing markedly improved brain tissue morphology compared with the model group (Supplementary Fig. S12A–C). Consistently, Nissl staining demonstrated disorganized hippocampal neuronal layers, reduced or blurred Nissl bodies, and abnormal cell morphology in the model group, reflecting marked neuronal injury. KPBIT@NPs treatment restored clearer neuronal layering, more abundant Nissl bodies, and a more regular cell arrangement, indicating effective alleviation of neuronal structural damage (Fig. 8D and K; Supplementary Fig. S13B). NeuN immunofluorescence staining was performed to assess mature neurons in the cortex and hippocampus as a marker of neuronal survival (Fig. 8E and L; Supplementary Fig. S13C). The model group showed reduced NeuN^+^ cells, indicative of neuronal loss or dysfunction. KPBIT@NPs significantly increased NeuN^+^ cell counts, with greater improvement than the other treatment groups, further supporting its neuroprotective effect. Golgi staining revealed that dendritic structures in model mice were reduced and simplified, indicating impaired neuronal plasticity. KPBIT@NPs treatment restored dendritic length and branching, suggesting protection and structural recovery of neuronal network architecture (Fig. 8M).

TEM analysis of mitochondrial ultrastructure showed that control mice had abundant, evenly distributed mitochondria with intact outer membranes and well-organized inner membrane cristae. In the model group, mitochondria were swollen, with disrupted or sparse cristae and compromised membrane integrity, indicating significant mitochondrial injury. KPBIT@NPs treatment markedly improved mitochondrial morphology, with clearer and more intact cristae and overall structure closer to that of controls, outperforming the other treatment groups, suggesting effective mitigation of AD-related mitochondrial damage and improved mitochondrial structural integrity (Fig. 8N). In summary, KPBIT@NPs significantly improve spatial learning and memory as well as daily functional behavior in AD model mice, while reducing Aβ deposition and preserving neuronal structure, survival, and mitochondrial integrity.

### 2.12 KPBIT@NPs reverse neuroinflammatory and metabolic imbalance and restore energy homeostasis in the brains of AD mice

**Fig. 9:**
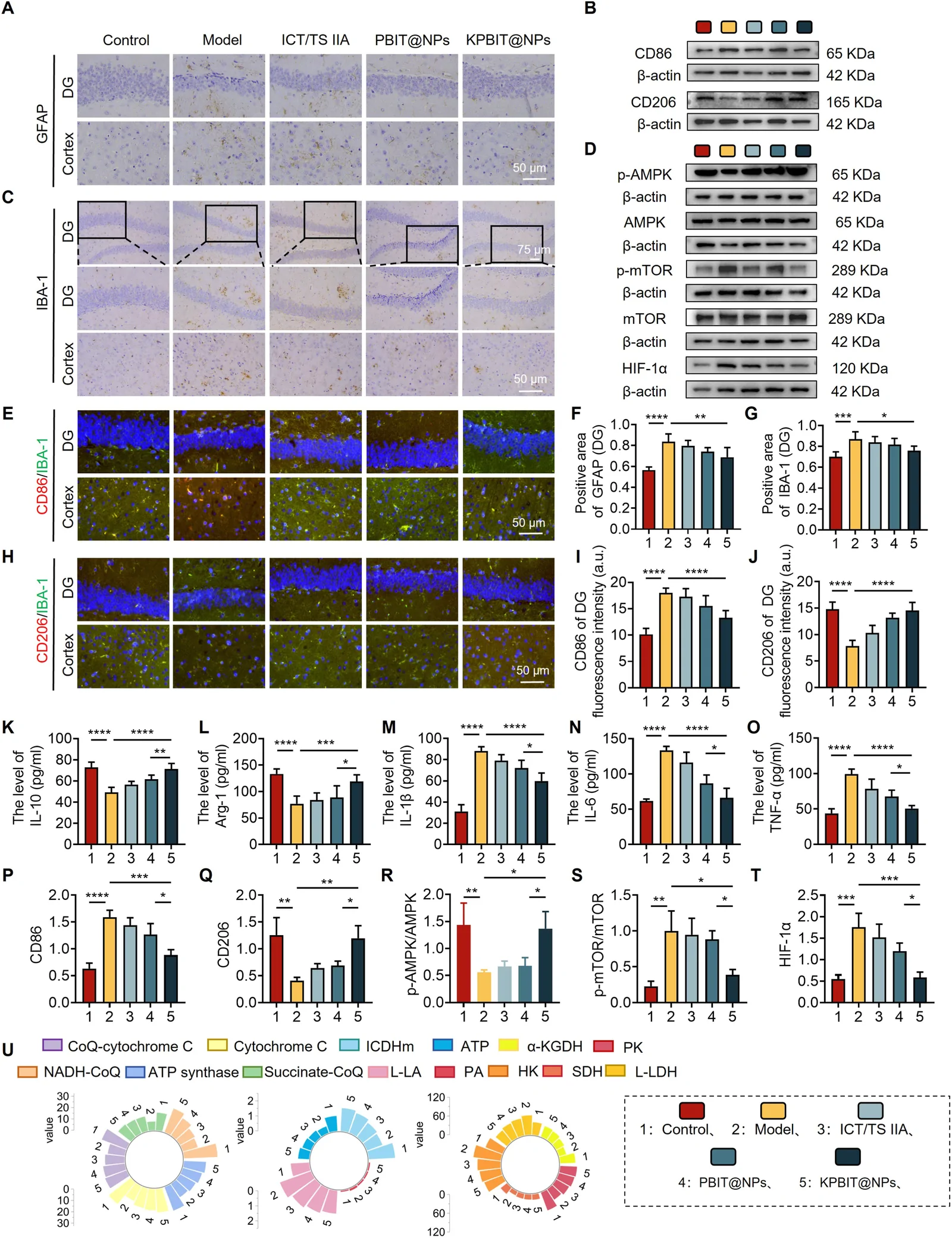
**Nanoformulations remodel the neuroinflammatory microenvironment and microglial polarization, with associated metabolic alterations, in APP**/PS1 mice. (A) Immunohistochemical staining of GFAP (astrocyte activation marker) in the cortex and hippocampal DG, Scale bar, 50 μm. (B) Western blot analysis of CD86 and CD206 protein expression in brain tissues. (C) Immunohistochemical staining of IBA-1 (microglial marker), Scale bar, 75 μm, Scale bar, 50 μm. (D) Western blot analysis of AMPK, mTOR, and HIF-1α protein expression. (E) Immunofluorescence staining of CD86/IBA-1 (M1 phenotype marker), Scale bar, 50 μm. (F) Semi-quantitative analysis of GFAP-positive area (n = 6). (G) Semi-quantitative analysis of IBA-1-positive area (n = 6). (H) Immunofluorescence staining of CD206/IBA-1 (M2 phenotype marker), Scale bar, 50 μm. (I) Semi-quantitative analysis of CD86 fluorescence intensity (n = 6). (J) Semi-quantitative analysis of CD206 fluorescence intensity (n = 6). (K–L) Expression levels of anti-inflammatory factors (IL-10 and Arg-1) (n = 6). (M − O) Expression levels of pro-inflammatory cytokines (IL-1β, IL-6, and TNF-α) (n = 6). (P–Q) Quantification of protein expression corresponding to (B) (n = 3). (R–T) Quantification of protein expression corresponding to (D) (n = 3). (U) Mitochondria-related metabolic assays in brain tissues, indicating global metabolic alterations in the brain (n = 6). Data are presented as mean ± SD (*P < 0.05, **P < 0.01, ***P < 0.001, ****P < 0.0001).

To determine whether the metabolic reprogramming effects observed in the BV2 inflammatory model in vitro could also be recapitulated in vivo, we systematically evaluated the regulatory effects of KPBIT@NPs on neuroinflammation, energy sensing, and metabolic function at the whole-brain tissue level in AD mice. Immunohistochemical staining was performed to assess astrocyte and microglial activation in brain tissues, using glial fibrillary acidic protein (GFAP) and ionized calcium-binding adapter molecule 1 (IBA-1) as markers, respectively. The model group exhibited markedly increased GFAP and IBA-1 immunoreactivity, indicating pronounced activation of astrocytes and microglia under AD pathological conditions. In contrast, treatment with KPBIT@NPs significantly reduced the immunoreactivity of both GFAP and IBA-1. This effect was more pronounced than that observed in the free-drug and non-targeted formulation groups, suggesting that KPBIT@NPs effectively suppress aberrant glial activation at the tissue level and alleviate neuroinflammatory burden in the brain (Fig. 9A and F; Fig. 9C and G; Supplementary Fig. S14A and S14B). To further elucidate the modulatory effects of KPBIT@NPs on microglial functional phenotypes, immunofluorescence co-staining of CD86, CD206, and IBA-1 was performed to distinguish pro-inflammatory and anti-inflammatory microglial states. In the model group, IBA-1-positive microglia showed markedly elevated CD86 expression and relatively low CD206 expression, indicating a predominance of the pro-inflammatory M1-like phenotype (Fig. 9E and I; Fig. 9H and J; Supplementary Fig. S14C and S14D). Following KPBIT@NPs treatment, CD86 expression in IBA-1-positive cells was significantly decreased, whereas CD206 expression was markedly enhanced. These results indicate a phenotypic shift of microglia from the pro-inflammatory M1 state toward the anti-inflammatory M2 state. This reprogramming pattern was highly consistent with the findings from BV2 cell experiments in vitro, further demonstrating that KPBIT@NPs can remodel microglial functional states in vivo.

To further validate the immunofluorescence findings at the protein level, Western blot analysis was performed on brain tissues to quantify the expression of CD86 and CD206. The results showed that CD86 expression was significantly elevated, whereas CD206 was markedly reduced in the model group. Upon treatment with KPBIT@NPs, CD86 expression was significantly downregulated, while CD206 was notably upregulated, with greater changes than those observed in other treatment groups (Fig. 9B and 9P–Q). These findings further confirm at the protein level that KPBIT@NPs effectively drive the phenotypic reprogramming of microglia from a pro-inflammatory to an anti-inflammatory state. We next assessed the levels of inflammatory mediators in brain tissues. Compared with the model group, KPBIT@NPs treatment significantly increased the expression of anti-inflammatory factors, including IL-10 and Arg-1 (Fig. 9K and L), while markedly decreasing the levels of pro-inflammatory cytokines such as IL-1β, IL-6, and TNF-α (Fig. 9M − O). Collectively, these results indicate that KPBIT@NPs not only suppress aberrant glial activation at the global level but also regulate microglial phenotypic balance at the functional level. This dual modulation effectively remodels the neuroinflammatory microenvironment and may contribute to neuronal survival and functional recovery.

Building on these favorable in vitro findings, we next systematically evaluated cerebral energy metabolism in whole-brain homogenates, focusing on tricarboxylic acid (TCA) cycle enzyme activities, mitochondrial OXPHOS, and glycolysis-associated enzymes and metabolites. Compared with the control group, the APP/PS1 model group exhibited significantly reduced activities of key TCA-cycle enzymes, including mitochondrial isocitrate dehydrogenase (ICDHm), α-ketoglutarate dehydrogenase (α-KGDH), and succinate dehydrogenase (SDH). In parallel, the activities of mitochondrial respiratory-chain complexes I–IV and ATP synthase (complex V) were markedly reduced, accompanied by decreased ATP content (Fig. 9U; Supplementary Fig. S15). These alterations are consistent with impaired mitochondrial OXPHOS and diminished cerebral bioenergetic capacity in APP/PS1 mice [69,70]. Concomitantly, the model group showed increased lactate and pyruvate levels and elevated activities of hexokinase (HK) and lactate dehydrogenase (L-LDH), whereas pyruvate kinase (PK) and PDH activities were reduced. This metabolic profile suggests the accumulation of glycolysis-associated metabolites and impaired pyruvate oxidation, consistent with a shift away from mitochondrial oxidative metabolism toward greater reliance on glycolysis-associated pathways. Treatment with free ICT + TS IIA or PBIT@NPs partially restored TCA-cycle and respiratory-chain enzyme activities, increased ATP content, and reduced the accumulation of glycolysis-associated metabolites. KPBIT@NPs produced the most pronounced overall normalization, particularly in SDH, α-KGDH, and ATP synthase activities, as well as lactate and pyruvate levels and HK and L-LDH activities (Fig. 9U; Supplementary Fig. S15). Collectively, these findings suggest that the targeted nanodelivery system more effectively restores oxidative metabolism and attenuates aberrant glycolytic remodeling in the brains of APP/PS1 mice.

We further investigated the molecular mechanisms underlying this metabolic reprogramming. Consistent with the in vitro observations, the AMPK-mTOR/HIF-1α signaling axis played a critical role in this process. Western blot analysis revealed that, compared with the control group, the model group showed significantly decreased p-AMPK levels and markedly increased p-mTOR and HIF-1α expression in brain tissues. After treatment, both the free-drug and PBIT@NPs groups partially reversed these signaling abnormalities, as evidenced by increased p-AMPK levels and decreased p-mTOR and HIF-1α expression (Fig. 9D and 9R–T). Importantly, KPBIT@NPs exerted the most pronounced regulatory effects, indicating a more effective restoration of the energy sensing–metabolic regulatory axis. Mechanistically, activation of AMPK suppresses mTOR signaling, whereas downregulation of HIF-1α reduces the aberrant expression of glycolysis-related genes. This coordinated regulation promotes a metabolic shift from glycolysis back to OXPHOS, thereby supporting energy homeostasis and alleviating inflammatory responses in brain tissues.

Taken together, in addition to its potent ability to regulate energy metabolism in microglia in vitro, KPBIT@NPs also enhance mitochondrial oxidative metabolism and suppress aberrant glycolysis at the whole-brain level in vivo, thereby correcting metabolic dysfunction under AD pathological conditions. On one hand, the formulation restores the balance of the AMPK-mTOR/HIF-1α axis, contributing to the recovery of energy-sensing pathways and suppression of inflammation-associated transcriptional programs. On the other hand, by restoring TCA cycle activity and respiratory chain function, KPBIT@NPs significantly increase ATP production in brain tissues. Compared with the free-drug and non-targeted formulations, KPBIT@NPs demonstrate superior efficacy across multiple metabolic parameters, highlighting their enhanced in vivo metabolic regulatory capacity. At the whole-brain level, the observed alterations in signaling proteins and metabolic parameters collectively indicate that KPBIT@NPs effectively restore metabolic homeostasis by reprogramming the AMPK-mTOR/HIF-1α signaling axis. In conjunction with the reduced expression of glial activation markers and the improved IBA-1/CD86 and IBA-1/CD206 co-staining profiles, these findings suggest that KPBIT@NPs effectively modulate the neuroimmune microenvironment, which may contribute to attenuation of neuroinflammation and restoration of metabolic balance within brain tissues.

Our in vitro BV2 microglia and in vivo AD mouse brain tissue data confirm that KPBIT@NPs activation of AMPK and inhibition of the mTOR/HIF-1α axis to interrupt the mutual exacerbation of neuroinflammation and pathological glycolysis, conferring neuroprotective effects. Nevertheless, our cerebral metabolic analyses were performed on bulk whole-brain homogenates, which cannot distinguish metabolic changes intrinsic to microglia from those in neurons, astrocytes or peripheral immune cells. Accordingly, we lack direct single-cell evidence to confirm microglia-specific metabolic reprogramming triggered by KPBIT@NPs in vivo, which is a key limitation to be addressed in future work. We plan a three-stage follow-up study to fill this gap. First, single-cell multi-omics coupled with spatial metabolic imaging will be applied to resolve microglial metabolic heterogeneity and define single-cell-resolution shifts in glycolysis and mitochondrial metabolism upon KPBIT@NPs treatment. Second, primary microglia purified from mouse brains will be subjected to extracellular flux assays, stable isotope tracing and mitochondrial stress tests to quantify microglia-exclusive TCA, glycolysis and OXPHOS activity changes. Third, microglia-specific genetic or pharmacological blockade of the AMPK–mTOR/HIF-1α axis will clarify whether this signaling pathway is required for KPBIT@NPs-mediated metabolic remodeling and neuroprotection, thus verifying its cell-autonomous causal role in microglia.

### 2.13 Biosafety evaluation

Following the systematic validation of the in vitro mechanisms and in vivo therapeutic efficacy of KPBIT@NPs, a biosafety assessment was performed to evaluate their feasibility as a potential neurodelivery platform. The evaluation included histological analysis of major organs in vivo, hemolysis assays, cytotoxicity tests, and peripheral blood hematological analysis (Supplementary Fig. S16). Histopathological examination of major organs, including the heart, liver, spleen, lung, and kidney, was conducted using H&E staining (Supplementary Fig. S16A). The results showed well-preserved tissue architecture with no obvious abnormalities in cellular morphology across all treatment groups. No evident inflammatory infiltration, necrosis, or pathological damage was observed. These findings were comparable to those of the control group, indicating that KPBIT@NPs did not induce detectable tissue toxicity under the experimental conditions. Hemocompatibility was further evaluated using a hemolysis assay to assess the interaction between KPBIT@NPs and erythrocytes. Within the tested concentration range, the hemolysis rates of KPBIT@NPs remained below the commonly accepted threshold for hemocompatibility and showed no significant difference compared with the negative control group. These results indicate that KPBIT@NPs exhibit favorable blood compatibility and did not induce appreciable erythrocyte disruption under the tested conditions (Supplementary Fig. S16B). Cytotoxicity assays were performed to determine the direct effects of KPBIT@NPs on BV2 and HT22 cells (Supplementary Fig. S16C and S16D). The results indicated that KPBIT@NPs did not significantly reduce cell viability across the tested concentrations. High levels of cell viability were maintained, indicating favorable cytocompatibility in vitro. To further evaluate systemic safety in vivo, peripheral blood hematological parameters were analyzed, including red blood cells, white blood cells, lymphocytes, and platelet-related indices. All measured parameters remained comparable to those of the control group and showed no significant abnormalities. These findings suggest that KPBIT@NPs did not cause obvious hematological toxicity under the tested conditions (Supplementary Fig. S16H–S16L).

To further evaluate whether KPBIT@NPs elicited peripheral immune activation, the serum levels of representative inflammatory cytokines were examined. No significant differences in TNF-α, IL-1β, and IL-10 levels were observed between the KPBIT@NP-treated group and the control group (Supplementary Fig. S16E–G). Notably, KPBIT@NPs did not increase the circulating levels of the major pro-inflammatory cytokines. These findings indicate that KPBIT@NPs did not induce detectable peripheral inflammatory activation under the present dosing regimen, further supporting their favorable short-term systemic immunocompatibility.

Although the biosafety results in this study are encouraging and no obvious short-term toxicity was observed, our current evaluation was limited to a relatively short observation period. Therefore, the long-term in vivo fate of KPBIT@NPs remains an important issue for future investigation. In particular, it will be necessary to further clarify their pharmacokinetic behavior, possible tissue retention, biodegradation, and clearance after prolonged or repeated administration. For KLV-modified polysaccharide nanoparticles, these questions are especially relevant because surface modification and repeated dosing may influence their distribution, persistence, and interaction with the immune system. In future work, we will further examine the long-term biodistribution and clearance of KPBIT@NPs in major organs, including the brain, liver, spleen, kidney, and other clearance-related tissues. We will also assess their degradation process in vivo and determine whether repeated administration leads to delayed toxicity or immune-related responses. These studies will help us better understand the long-term safety profile and translational potential of KPBIT@NPs.

## 3 Conclusion

In summary, inspired by the TCM principle of “BuShen HuoXue,” this study developed KPBIT@NPs, a multifunctional brain-targeted nanotherapeutic platform based on engineered PNP, for the synergistic delivery of ICT and TS IIA. By integrating a natural polysaccharide backbone, ROS-responsive phenylboronic ester linkages, KLV-mediated BBB penetration, and activated microglia targeting, this platform enables pathological microenvironment-responsive drug release and microglia-directed immunometabolic intervention in AD. KPBIT@NPs reactivate AMPK and suppress the mTOR/HIF-1α axis, thereby alleviating pathological glycolytic skewing, restoring mitochondrial oxidative metabolism, and promoting microglial remodeling toward a reparative phenotype, which collectively contributes to their therapeutic effects in AD models. This study proposes a polysaccharide-based nanotherapeutic strategy that integrates natural product co-delivery with microglial immunometabolic reprogramming, providing a potential intervention framework for neurodegenerative diseases driven by coupled metabolic–inflammatory dysregulation. Nevertheless, further studies are needed to define the cell-specific metabolic effects, long-term in vivo fate, and translational safety of KPBIT@NPs in more advanced preclinical models.

## 4 Materials and methods

Materials and methods are described in the Supplementary.

## CRediT authorship contribution statement

**Ge Zhang:** Writing – original draft, Methodology, Conceptualization. **Liang Kong:** Supervision, Conceptualization. **Rui-bo Guo:** Investigation. **Shuai-wen Ding:** Methodology. **Yang Liu:** Formal analysis. **Juan Zang:** Investigation. **Ying Zheng:** Validation. **Bin Wei:** Visualization. **Zhi-chao Chen:** Methodology. **Ying Yang:** Writing – review & editing, Writing – original draft, Methodology. **Xue-tao Li:** Writing – review & editing, Writing – original draft, Methodology, Funding acquisition. **Yang Yu:** Writing – review & editing, Writing – original draft, Methodology, Funding acquisition.

## Funding

This work was supported by the 10.13039/501100001809National Natural Science Foundation of China (Grant No. 82404867 and 82574866), Liaoning Provincial Science and Technology Program Joint Plan (Grant No. 2025-MSLH-479 and 2024-MSLH-289), Specialized Project for the Cultivation of Outstanding Young and Middle-aged Science and Technology Talents in Shenyang (Grant No. RC240317), Liaoning Young Elite Scientists Sponsorship Program, the 10.13039/100007452Wu Jieping Medical Foundation Special Research Fund (Grant No. 320.6750.2024-18-60).

## Declaration of competing interests

There are no conflicts to declare.

## Data Availability

Data will be made available on request.
