# Supplementary material for: Microglia-targeted Panax notoginseng polysaccharide nanoparticles alleviate Alzheimer's disease via AMPK-mTOR/HIF-1α-mediated immunometabolic reprogramming
Source: Mater Today Bio. 2026 Aug 28;40:103620. doi: 10.1016/j.mtbio.2026.103620 (PMC13545451; doi:10.1016/j.mtbio.2026.103620)
Supplement: Supplementary file 1 [file mmc1.docx]

**Supplementary**

**Fig. S1. Electron microscopy characterization of KPBIT@NPs morphology.** (A) Representative scanning electron microscopy image of KPBIT@NPs. Scale bar, 50 nm. (B) Representative transmission electron microscopy image of KPBIT@NPs. Scale bar, 100 nm.

**Fig. S2.** **In vitro cumulative release behavior of tanshinone IIA (TS IIA) from KPBIT@NPs nanoparticles.** Cumulative release profile of TS IIA from KPBIT@NPs incubated in PBS, 10% fetal bovine serum (FBS), and H_2_O_2_ solutions with gradient concentrations (0.1, 1, 10, 100 μM, and 1 mM) (n = 3).

**Fig. S3. Cellular uptake of different formulations in bEnd.3 and BV2 cells assessed by flow cytometry.** (A) Flow cytometric analysis of uptake in AD-bEnd.3 cells. (B) Flow cytometric analysis of uptake in AD-BV2 cells. (C) Quantification of fluorescence intensity corresponding to (A). (D) Quantification of fluorescence intensity corresponding to (B). Data are presented as mean ± SD (^*^*P* < 0.05, ^**^*P* < 0.01, ^***^*P* < 0.001, ^****^*P* < 0.0001) (n = 3).

**Fig. S4. Validation of the integrity of the in vitro blood–brain barrier (BBB) model. Transendothelial electrical resistance (TEER), sodium fluorescein (Na-FLU) permeability, and immunofluorescence staining of tight junction proteins (ZO-1, Occludin, F-actin) and the injury marker VEGF were detected to evaluate BBB barrier function.** (A) Quantification of TEER values in the Control and AD-bEnd.3 groups. (B) Quantitative analysis of the apparent permeability coefficient (Papp) of Na-FLU across bEnd.3 monolayers. (C–F) Representative immunofluorescence micrographs of bEnd.3 monolayers from Control and AD-bEnd.3 groups. Cell nuclei were counterstained with Hoechst 33342; F-actin was stained by phalloidin, while Occludin, ZO-1 and VEGF were visualized via immunolabeling. Scale bar, 75 μm. (G–J) Semi-quantitative measurement of mean fluorescence intensity for phalloidin, Occludin, ZO-1, and VEGF, respectively. Data are presented as mean ± SD (^*^*P* < 0.05, ^**^*P* < 0.01, ^***^*P* < 0.001, ^****^*P* < 0.0001) (n = 3).

**Fig. S5. Magnified local view of the brain in Figure 2J from in vivo imaging.** Magnified brain area extracted from the whole-body fluorescence image in Fig. 2J.

**Fig. S6. Flow cytometric analysis of CD86 and CD206 expression in BV2 cells.** (A) CD86 expression. (B) CD206 expression. (C, D) Quantification of fluorescence intensity corresponding to (A, B). Data are presented as mean ± SD (^*^*P* < 0.05, ^**^*P* < 0.01, ^***^*P* < 0.001, ^****^*P* < 0.0001) (n = 3).

**Fig. S7. Flow cytometric analysis of apoptosis in HT22 cells treated with conditioned media under different conditions.** (A) Representative apoptosis profiles. (B) Quantification of apoptosis rates. Data are presented as mean ± SD (^*^*P* < 0.05, ^**^*P* < 0.01, ^***^*P* < 0.001, ^****^*P* < 0.0001) (n = 3).

**Fig. S8. Live/dead staining analysis of HT22 cells treated with conditioned media under different conditions.** (A) Representative fluorescence images based on Calcein-AM/PI staining. (B) Quantification of cell viability corresponding to (A). Data are presented as mean ± SD (^*^*P* < 0.05, ^**^*P* < 0.01, ^***^*P* < 0.001, ^****^*P* < 0.0001) (n = 3) (ns, not significant, *P* ≥ 0.05).

**Fig. S9. Levels of inflammatory cytokines in BV2 cell culture supernatants after treatment with different formulations.** (A–C) Pro-inflammatory cytokines (IL-6, TNF-α, IL-1β). (D–E) Anti-inflammatory factors (Arg-1 and IL-10). Groups: 1. Control; 2. Model; 3. KPB@NPs; 4. ICT/TS IIA; 5. PBIT@NPs; 6. KPBIT@NPs. Data are presented as mean ± SD (^*^*P* < 0.05, ^**^*P* < 0.01, ^***^*P* < 0.001, ^****^*P* < 0.0001) (n = 3).

**Fig. S10. Supplementary quantitative analysis of BV2 microglial morphology and polarization phenotype.** (A) Semi-quantitative analysis of BV2 cellular circularity calculated from bright-field micrographs (Fig. 3G). (B) Semi-quantitative analysis of the average process length of BV2 cells calculated from bright-field micrographs (Fig. 3G). (C) Flow cytometric quantification of CD86 MFI, derived from flow data in Fig. 3H. (D) Flow cytometric quantification of CD206 MFI, derived from flow data in Fig. 3K. Data are presented as mean ± SD (^*^*P* < 0.05, ^**^*P* < 0.01, ^***^*P* < 0.001, ^****^*P* < 0.0001) (n = 3).

**Fig. S11. Network pharmacology analysis.** (A) Venn diagram of disease-related and TCM-related targets. (B) TCM-active ingredient-core target-disease interaction network. (C) GO enrichment analysis of intersecting targets.

**Fig. S12. H&E staining of mouse cerebral sections.** (A) Representative H&E-stained images of brain tissue sections from mice treated with different formulations. Scale bar, 50 μm. (B) Quantification of the percentage of damaged neurons in the hippocampal DG, calculated from H&E images in (A). (C) Quantification of the percentage of damaged neurons in the cerebral cortex, calculated from H&E images in (A). Data are presented as mean ± SD (^*^*P* < 0.05, ^**^*P* < 0.01, ^***^*P* < 0.001, ^****^*P* < 0.0001) (n = 6).

**Fig. S13.** **Quantitative analysis of cortical Aβ plaques, Nissl bodies and NeuN fluorescence intensity.** (A) Semi-quantitative analysis of Aβ plaques in the cortical region corresponding to Fig. 8C. (B) Semi-quantitative analysis of Nissl bodies in the cortical region corresponding to Fig. 8D. (C) Semi-quantitative analysis of NeuN fluorescence intensity in the cortical region corresponding to Fig. 8E. Data are presented as mean ± SD (^*^*P* < 0.05, ^**^*P* < 0.01, ^***^*P* < 0.001, ^****^*P* < 0.0001) (n = 6).

**Fig. S14. Semi-quantitative analysis of neuroinflammatory markers in the cerebral cortex.** (A) Semi-quantitative analysis of GFAP-positive area in the cortex, derived from immunohistochemical images in Fig. 9A. (B) Semi-quantitative analysis of IBA-1-positive area in the cortex, derived from immunohistochemical images in Fig. 9C. (C) Semi-quantitative analysis of the MFI of CD86 in the cortex, corresponding to dual immunofluorescence images in Fig. 9E. (D) Semi-quantitative analysis of the MFI of CD206 in the cortex, corresponding to dual immunofluorescence images in Fig. 9H. Data are presented as mean ± SD (^*^*P* < 0.05, ^**^*P* < 0.01, ^***^*P* < 0.001, ^****^*P* < 0.0001) (n = 6).

**Fig. S15.** **Nanoformulations restore mitochondrial respiratory-chain activity and energy metabolism in the brain tissue of APP/PS1 mice.** (A–E) Activities of mitochondrial respiratory-chain complexes I–V: NADH–coenzyme Q (CoQ) reductase, complex I (A); succinate–CoQ reductase, complex II (B); CoQ–cytochrome c reductase, complex III (C); cytochrome c oxidase, complex IV (D); and ATP synthase, complex V (E). (F–I) Activities of α-ketoglutarate dehydrogenase (α-KGDH) (F), succinate dehydrogenase (SDH) (G), pyruvate kinase (PK) (H), and pyruvate dehydrogenase (PDH) (I). (J–O) Pyruvate content (J), L-LDH activity (K), L-lactate content (L), mitochondrial isocitrate dehydrogenase (ICDHm) activity (M), hexokinase (HK) activity (N), and total ATP content (O). Groups: 1, Control; 2, Model; 3, free ICT + TS IIA; 4, PBIT@NPs; and 5, KPBIT@NPs. Data are presented as mean ± SD. Data are presented as mean ± SD from n = 6 biologically independent mice per group. ^*^*P* < 0.05, ^**^*P* < 0.01, ^***^*P* < 0.001, ^****^*P* < 0.0001.

**Fig. S16. Biosafety evaluation of nanoformulations.** (A) Representative H&E-stained images of major organs (heart, liver, spleen, lung, and kidney) (n = 6, Scale bar, 50 μm). (B) Hemolysis assay (n = 3). (C) Cytotoxicity in BV2 cells (n = 8). (D) Cytotoxicity in HT22 cells (n = 8). (E–G) Peripheral-blood levels of TNF-α, IL-10, and IL-1β, respectively, in mice after the indicated treatments (n = 3). (H–L) Hematological analysis, including white blood cells, red blood cells, lymphocytes, hemoglobin, and platelets (n = 3). Data are presented as mean ± SD (ns, not significant, *P* ≥ 0.05)

**Table S1.** Self-assembly properties of PNP-CPBA conjugates synthesized under different conditions (n = 3)

| **Entry** | **CPBA (mmol)** | **PNP (mg)** | **CPBA conjugation content (%)** | **CAC (mg/mL)** | **Diameter (nm)** | **PDI** | **Diameter** |
| --- | --- | --- | --- | --- | --- | --- | --- |
| 1 | 0.01 | 400 | 0.87% | 0.697 | 62.90 ± 0.121 | 0.206 ± 0.00557 |  |
| 2 | 0.05 | 400 | 0.94% | 0.615 | 63.26 ± 0.172 | 0.213 ± 0.00709 |  |
| 3 | 0.10 | 400 | 2.33% | 0.396 | 63.63 ± 0.218 | 0.203 ± 0.00436 |  |
| 4 | 0.25 | 400 | 4.17% | 0.187 | 63.97 ± 0.0569 | 0.212 ± 0.0155 |  |
| 5 | 0.50 | 400 | 8.95% | 0.0358 | 64.64 ± 0.327 | 0.210 ± 0.0107 |  |
| 6 | 0.75 | 400 | 11.73% | Not detected^1^ | - | - | - |
| 7 | 1.00 | 400 | 13.61% | Not detected^1^ | - | - | - |

^1^ No distinct CAC was detected, which can be attributed to the poor water solubility of the conjugate. As a result, the formation of stable micellar assemblies was hindered.

**Table S2.** Encapsulation efficiency (EE) of ICT and TS IIA in KPBIT@NPs (n = 3)

| **NPs** | **Loaded Drug** | **EE values of individual drugs** | **Mean ± SD** |
| --- | --- | --- | --- |
| KPBIT@NPs | ICT | 92.23%  94.12%  89.95% | (92.10 ± 2.09)% |
|  | TS IIA | 87.87%  89.21%  85.40% | (87.49 ± 1.93)% |

**Table S3.** Particle size, polydispersity index (PDI), and zeta potential of the prepared nanoparticles (n = 3)

|  | **Diameter (nm)** | **PDI** | **Zeta (mV)** |
| --- | --- | --- | --- |
| KPB@NPs | 66.32 ± 0.826 | 0.216 ± 0.0304 | -1.80 ± 0.40 |
| PBIT@NPs | 76.39 ± 0.717 | 0.184 ± 0.0951 | -1.40 ± 0.20 |
| KPBIT@NPs | 79.33 ± 0.191 | 0.0593 ± 0.0442 | -2.10 ± 0.55 |

**Table S4.** Stability of KPBIT@NPs under different conditions (n = 3)

| KPBIT@NPs | | 1 d | 2 d | 4 d | 6 d | 8 d | 10 d | 12 d | 14 d |
| --- | --- | --- | --- | --- | --- | --- | --- | --- | --- |
| 4℃ | Diameter (nm) | 79.51 ± 1.62 | 80.14 ± 2.62 | 81.56 ± 1.40 | 81.91 ± 1.54 | 82.10 ± 1.65 | 85.24 ± 2.37 | 88.02 ± 1.69 | 90.27 ± 0.45 |
|  | PDI | 0.158 ± 0.00323 | 0.141 ± 0.00325 | 0.157 ± 0.00912 | 0.141 ± 0.00183 | 0.136 ± 0.00349 | 0.112 ± 0.00963 | 0.201 ± 0.00132 | 0.197 ± 0.00115 |
| FBS | Diameter (nm) | 83.14 ± 8.28 | 87.62 ± 6.56 | 94.37 ± 2.56 | 95.48 ± 1.34 | 95.84 ± 0.74 | 97.98 ± 2.47 | 98.64 ± 3.90 | 100.26 ± 0.37 |
|  | PDI | 0.146 ± 0.00716 | 0.185 ± 0.00731 | 0.212 ± 0.00260 | 0.199 ± 0.00500 | 0.236 ± 0.00159 | 0.193 ± 0.00524 | 0.100 ± 0.00209 | 0.133 ± 0.00270 |

**Table S5.** Binding free energy of ICT and TS IIA to target proteins (n = 3)

| **Compound** | **Target Protein** | **Binding Energy(ΔG, kcal/mol)** | | | **AVERAGE ± SD** |
| --- | --- | --- | --- | --- | --- |
|  |  | **1** | **2** | **3** |  |
| ICT | AMPK | -8.1 | -8.2 | -8.2 | -8.17 ± 0.05 |
| ICT | mTOR | -6.8 | -6.8 | -7.9 | -7.17 ± 0.52 |
| TS IIA | AMPK | -10.1 | -8.8 | -10.2 | -9.70 ± 0.64 |
| TS IIA | mTOR | -9.6 | -9.2 | -7.7 | -8.83 ± 0.82 |

**Materials and Methods**

**1 Materials, cells, and animals**

Tanshinone IIA (TS IIA) and Icaritin (ICT), with purity ≥ 98%, were procured from Chengdu Purfield Biotechnology Co., Ltd. (Chengdu, China). Water-soluble *Panax notoginseng* polysaccharide were obtained from Shanxi Yuzhou Biotechnology Co., Ltd. (Shanxi, China). Deoxyglucose (2-DG), glucose, oligomycin, carbonyl cyanide 4-(trifluoromethoxy)phenylhydrazone (FCCP), rotenone (ROT), and antimycin A (AA), 4-carboxyphenylboronic acid pinacol ester (CPBA), 4-dimethylaminopyridine (DMAP), 1-ethyl-3-(3-dimethylaminopropyl)carbodiimide hydrochloride (EDC·HCl), lipopolysaccharide (LPS), and bovine serum albumin (BSA) were purchased from Shanghai Aladdin Biochemical Technology Co., Ltd. (Shanghai, China). β-amyloid protein (1-42) (β-Amyloid, Aβ_1-42_) was purchased from Zhejiang Hongtuo Biotechnology Co., Ltd. (Zhejiang, China). The KLVFFAED peptide was acquired from Xi’an Ruixi Biotechnology Co., Ltd. (Xi’an, China). Neutral balsam, hematoxylin and eosin (H&E) staining kit, and Nissl staining kit were purchased from Dalian Meilun Biotechnology Co., Ltd. (Dalian, China). 1,1’-Dioctadecyl-3,3,3’-trimethylindotricarbocyanine iodide (DIR) and Coumarin 6 (Cou-6) were obtained from Jiangsu Kaiji Biotechnology Co., Ltd. (Jiangsu, China). Reactive oxygen species (ROS) detection kit, live/dead staining kit (Calcein-AM/PI), and mitochondrial membrane potential assay kit (JC-1) were supplied by Beijing Beyotime Biotechnology Co., Ltd. (Beijing, China). The following kits were acquired from Beijing Solarbio Science & Technology Co., Ltd. (Beijing, China): Sodium fluorescein (Na-FLU), Mitochondrial Complex I/NADH-CoQ Reductase Activity Assay Kit, Mitochondrial Complex II/Succinate-CoQ Reductase Activity Assay Kit, Mitochondrial Complex III/CoQ-Cytochrome c Reductase Activity Assay Kit, Mitochondrial Complex IV/Cytochrome c Oxidase Activity Assay Kit, Mitochondrial Complex V/ATP Synthase Activity Assay Kit, Mitochondrial Isocitrate Dehydrogenase Kit (ICDHm), Adenosine Triphosphate (ATP) Content Assay Kit, L-Lactate Content Kit (L-LA), Pyruvate Content Kit (PA), α-Ketoglutarate Dehydrogenase Kit (α-KGDH), L-Lactate Dehydrogenase Kit (L-LDH), Hexokinase Kit (HK), Succinate Dehydrogenase Kit (Complex II, SDH), Pyruvate Kinase Kit (PK), Pyruvate Dehydrogenase (PDH) Activity Assay Kit, and Enzyme-Linked Immunosorbent Assay (ELISA) Kits. Total superoxide dismutase (SOD) and malondialdehyde (MDA) assay kits were procured from Nanjing Jiancheng Bioengineering Institute. (Nanjing, China). Electrophoresis buffer, transfer buffer, luminescent substrate, and skim milk powder were obtained from Servicebio Biotechnology Co., Ltd. (Beijing, China). IBA-1 antibody was purchased from Fujifilm Wako Pure Chemical Corporation (Osaka, Japan). Antibodies against CD86, CD206, AMP-activated protein kinase (AMPK), phosphorylated AMPK (p-AMPK), mammalian target of rapamycin (mTOR), phosphorylated mTOR (p-mTOR), hypoxia-inducible factor 1α (HIF-1α), β-amyloid 1-42 (Aβ_1-42_), glial fibrillary acidic protein (GFAP), and neuronal nuclei (NeuN), Zonula Occludens-1 (ZO-1), Occludin and Vascular Endothelial Growth Factor (VEGF) as well as fluorescein isothiocyanate (FITC)-conjugated, CY3-conjugated, and horseradish peroxidase (HRP)-labeled secondary antibodies, were supplied by Boster Biological Technology Co., Ltd. (Wuhan, China). Phalloidin, Pyruvate dehydrogenase E1 subunit alpha (PDHE1α), phosphorylated PDHE1α (p-PDHE1α), 6-phosphofructo-2-kinase/fructose-2,6-bisphosphatase 3 (PFKFB3), lactate dehydrogenase A (LDHA), and ATP synthase F1 subunit alpha (ATP5A) were purchased from Servicebio Biotechnology Co., Ltd. (Beijing, China). All other reagents used were of analytical grade.

DMEM medium, trypsin, penicillin-streptomycin dual antibiotics, and fetal bovine serum were purchased from Gibco Life Sciences (USA). Mouse microglial cells (BV2 cells), mouse cerebral microvascular endothelial cells (bEnd.3 cells), and hippocampal neuronal cells (HT22 cells) were obtained from the Cell Bank of the Chinese Academy of Sciences (Shanghai, China). All three cell lines were cultured in an incubator maintained at 37°C in a humidified atmosphere of 95% air and 5% CO_2_.

Six-month-old male APP/PS1 mice and C57BL/6J mice were purchased from Beijing Huafukang Biotechnology Co., Ltd. (Beijing, China). All experimental procedures were conducted in compliance with the guidelines of the Experimental Animal Ethics Committee of Liaoning University of Traditional Chinese Medicine, and this study was approved by the said committee (Approval No. 210000620250228).

**2 Characterization**

^1^H NMR: Measurements were performed using a Bruker 500 MHz NMR spectrometer (Bruker, Germany) with samples dissolved in deuterated dimethyl sulfoxide (DMSO-*d_6_*). Fourier Transform Infrared Spectroscopy (FTIR): FTIR spectra were obtained using a Bruker Equinox 55 FTIR spectrometer (Bruker, Germany) in the range of 400–4000 cm^-1^. Samples were prepared by spreading them on KBr discs and drying them under infrared lamps for 2 min. Each spectrum was recorded by averaging 32 scans at a resolution of 4 cm-1 to enhance the signal-to-noise ratio and accuracy. Morphological changes before and after self-assembly: Morphological characteristics of PNP and PNP-CPBA were observed using a scanning electron microscope (SEM, Regulus 8100, Hitachi, Japan). Surface morphology of KPBIT@NPs was characterized by transmission electron microscopy (TEM, JEM-1200EX, JEOL Ltd., Tokyo, Japan). Dynamic Light Scattering (DLS): The Malvern Zetasizer Nano ZS90 instrument was used to analyze nanoparticle hydration size, polydispersity index (PDI), and Zeta potential. The encapsulation efficiencies of TS IIA and ICT in the formulation were determined separately by HPLC. For TS IIA, a Diamonsil C18 column (5 μm, 250 × 4.6 mm) was used with a mobile phase consisting of acetonitrile and water (70:30, v/v) at a flow rate of 1 mL/min. The eluent was monitored by a UV detector at 270 nm. For ICT, the same column was employed with a mobile phase of methanol and water (60:40, v/v) at a flow rate of 1 mL/min, and the absorbance was measured at 360 nm. Prior to analysis, free and encapsulated drugs were separated by ultrafiltration centrifugation. The encapsulated drug was released from the formulation by disrupting the nanoparticles with methanol. The encapsulation efficiency (*EE*%) was calculated using the following formula:

*Encapsulation Efficiency* (*EE*, %)=$\frac{\text{W}_{\text{Total drug}}\text{−}\text{W}_{\text{Free drug}}}{\text{W}_{\text{Total drug}}}$×100%

**3 Preparation of modified *Panax notoginseng polysaccharide* and drug-loaded nanoparticles**

The *Panax notoginseng* polysaccharide used in this study was purchased from Shaanxi Yuzhou Biotechnology Co., Ltd. In our previous study, the crude *Panax notoginseng* polysaccharide (PNP) was further purified and characterized as a homogeneous polysaccharide with a molecular weight of 3589.22 Da, composed of glucose, mannose, and rhamnose.

**3.1 Synthesis and optimization of CPBA-modified PNP (PNP-CPBA)**

PNP-CPBA conjugates were synthesized via carbodiimide-mediated esterification. Briefly, predetermined amounts of 4-carboxyphenylboronic acid pinacol ester (CPBA), 4-dimethylaminopyridine (DMAP), and 1-ethyl-3-(3-dimethylaminopropyl)carbodiimide hydrochloride (EDC·HCl) (as listed in Table S1) were added to a 100 mL three-necked round-bottom flask under nitrogen protection. Anhydrous dimethyl sulfoxide (DMSO) was added, and the mixture was stirred at 500 rpm for 0.5 h at room temperature to activate the carboxyl groups of CPBA. Meanwhile, 400 mg of PNP was dissolved in 10 mL of anhydrous DMSO with the aid of ultrasonication until completely dissolved. The PNP solution was then added dropwise to the activated CPBA solution, and the reaction was allowed to proceed under stirring at room temperature for 48 h. After completion, the reaction mixture was poured into 10 volumes of anhydrous ethanol and left overnight at 4°C to precipitate the crude product. The precipitate was collected by centrifugation (8,000 × g, 10 min), redissolved in distilled water, and dialyzed against distilled water using a dialysis bag (MWCO 3000 Da) for 48 h to remove unreacted reagents and by-products. The dialysate was finally lyophilized to obtain PNP-CPBA as a pale yellow solid.

To identify the conjugate with optimal self-assembly performance, a series of PNP-CPBA batches with different CPBA/PNP feed ratios (Table S1) were evaluated for their critical aggregation concentration (CAC), particle size, and polydispersity index (PDI) using dynamic light scattering (DLS) and a fluorescence probe technique. The batch exhibiting the lowest CAC, smallest particle size, and narrowest PDI was selected for subsequent peptide conjugation.

**3.2 Conjugation of KLVFFAED peptide to PNP-CPBA (KLV-PNP-CPBA)**

KLVFFAED peptide was conjugated to PNP-CPBA via a carbodiimide-mediated esterification reaction. Briefly, KLVFFAED peptide (100 mg) and PNP-CPBA (100 mg) were mixed at a mass ratio of 1:1, which had been optimized in preliminary experiments. The two reactants were dissolved in 10 mL of anhydrous DMSO, followed by the addition of EDC·HCl (19.17 mg) and DMAP (12.22 mg). The reaction mixture was continuously stirred under a nitrogen atmosphere at room temperature for 48 h. To minimize moisture-induced side reactions, all glassware was oven-dried prior to use, and anhydrous DMSO was employed throughout the procedure. Upon completion of the reaction, the mixture was transferred into dialysis tubing with a molecular weight cut-off (MWCO) of 3000 Da and dialyzed against distilled water at room temperature for 48 h to remove unreacted peptide, residual EDC·HCl and DMAP, and low-molecular-weight by-products. The dialyzed solution was subsequently lyophilized to obtain KLV-PNP-CPBA as a dry powder.

**3.3 Preparation of drug-loaded nanoparticles**

Blank nanoparticles were prepared by dissolving 100 mg of the carrier material (PNP-CPBA or KLV-PNP-CPBA) in 10 mL of phosphate-buffered saline (PBS, pH 7.4), followed by ultrasonication (40 W, 10 min) and extrusion twice through a 0.22 μm polycarbonate membrane.

For drug loading, ICT and TS IIA were co-dissolved in 2 mL of DMSO at a molar ratio of 1:1. The organic phase was added dropwise into the blank nanoparticle suspension under ultrasonication (40 W, ice bath). The mixture was continuously sonicated for 1 h to promote efficient drug encapsulation. The resulting dispersion was dialyzed against deionized water (MWCO 3000 Da) for 24 h to remove DMSO and free drugs. Subsequently, the nanoparticle suspension was extruded twice through a 0.22 μm polycarbonate membrane to obtain uniformly sized drug-loaded nanoparticles.

For imaging studies, fluorescent probe-labeled nanoparticles were prepared using the same procedure, where ICT and TS IIA were replaced with 1,1’-dioctadecyl-3,3,3’,3’-tetramethylindotricarbocyanine iodide (DIR) or coumarin 6 (Cou-6), yielding PBD@NPs (with DIR), PBC@NPs (with Cou-6), KPBD@NPs, and KPBC@NPs, respectively.

**4 Determination of critical aggregation concentration (CAC)**

Pyrene was employed as a fluorescence probe due to its high sensitivity to environmental polarity for CAC determination of nanoparticles. A 6 × 10^-4^ M pyrene stock solution in acetone was prepared, and 4 μL of this solution was added into clean glass vials. The acetone was allowed to evaporate overnight under light-protected conditions to remove residual solvent. Subsequently, 400 μL of nanoparticle solutions at various concentrations were added to the vials and allowed to equilibrate at room temperature overnight to facilitate the partitioning of pyrene into the hydrophobic cores of the nanoparticles. Fluorescence emission spectra were recorded from 300 to 400 nm with an excitation wavelength of 340 nm. The fluorescence intensities at 372 nm (I_1_) and 384 nm (I_3_) were measured, and the I_1_/I_3_ ratio was calculated to evaluate the microenvironment polarity of the nanoparticle cores. The CAC value was determined from the intersection point of two linear regions in the plot of I_1_/I_3_ versus nanoparticle concentration.

**5 In vitro release study**

Free ICT/TS IIA and KPBIT@NPs were incubated in PBS, PBS containing 10% FBS, and PBS supplemented with various concentrations of H_2_O_2_ (0.1 μM, 1 μM, 10 μM, 100 μM, and 1 mM) at 37°C for 30 h. A 2 mL aliquot of the formulation was loaded into a dialysis bag (MWCO 3000 Da) and immersed in 200 mL of the corresponding release medium under continuous stirring. At predetermined time intervals, 1 mL of the external release medium was withdrawn and replaced with an equal volume of fresh medium to maintain a constant volume. The concentrations of ICT and TS IIA were quantified by HPLC at 360 nm and 270 nm, respectively. The cumulative release under different conditions was calculated according to the following equation:

Cumulative release (%) = $\frac{\text{∑}_{\text{i}\text{=1}}^{\text{n}}\text{C}_{\text{i}}\text{V}\text{+}\text{C}_{\text{n}}\text{(}\text{V}_{\text{1}}\text{−}\text{nV}\text{)}}{\text{M}}\text{×}\text{100\%}$

Where *C_n_* (μg/mL) is the drug concentration at the nth sampling point; *V_1_* (mL) is the total volume of the release medium; *V* (mL) is the sampling volume; and *M* (mg) is the total amount of drug loaded in the formulation.

**6 Cell culture**

**6.1 Cell passaging**

BV2 cells were cultured in cell culture dishes and routinely observed under a microscope. The culture medium was replaced according to cell confluency and medium color. When cell confluency reached 80–90%, passaging was performed. The old medium was removed, and the cells were washed twice with sterile PBS. Fresh medium was then added, and the cells were gently pipetted to obtain a single-cell suspension. The cell suspension was transferred into centrifuge tubes, gently mixed, and seeded into new culture dishes, followed by incubation in a humidified incubator. bEnd.3 cells were cultured in cell culture flasks and routinely monitored. The medium was replaced based on cell growth status. When the confluency reached 80–90%, cells were passaged. After removing the medium, cells were washed twice with sterile PBS and digested with trypsin at 37°C until cell detachment was observed. Fresh medium was added to terminate digestion, and the cell suspension was collected and centrifuged at 300 × g for 3 min. The cell pellet was resuspended in fresh medium, gently pipetted to disperse evenly, and transferred into new culture flasks for further incubation. HT22 cells were passaged following the same procedure as described for bEnd.3 cells.

**7 Establishment of in vitro and in vivo models and dose determination**

**7.1 In vitro experiments**

Based on preliminary experiments and previous studies, an in vitro AD model was established as follows [1,2]. BV2 and bEnd.3 cells were stimulated with pre-aggregated Aβ_1-42_ (20 μM, incubated at 37°C for 24 h) and lipopolysaccharide (LPS, 2 μg/mL) for 12 h to induce neuroinflammatory and neurotoxic injury mimicking AD pathology. HT22 cells were treated with Aβ_1-42_ (20 μM) for 12 h to establish a neuronal injury model. Unless otherwise specified, this protocol was used for all in vitro experiments. BV2 cells were seeded in 96-well plates at a density of 1 × 10^4^ cells per well and cultured at 37°C in a humidified atmosphere containing 5% CO_2_. After cell attachment, cells were first exposed to Aβ_1-42_ and LPS for 12 h to induce injury, followed by treatment with TS IIA (5–50 μM) for an additional 12 h. Cell viability was measured using the CCK-8 assay, and absorbance was recorded at 450 nm. EC50 values were calculated using nonlinear regression analysis in GraphPad Prism. ICT was tested at concentrations ranging from 2 to 50 μM using the same procedure. Free drugs were dissolved in aqueous solution containing 1% Tween 80 as a solubilizing agent, and corresponding vehicle controls were included. All experiments were performed in triplicate and repeated at least three times independently. Subsequent studies demonstrated that the combination of ICT and TS IIA at a molar ratio of 1:1 produced the most pronounced pharmacological effects. Based on EC50 analysis and preliminary data, the final concentrations of ICT and TS IIA used in subsequent in vitro experiments were both set at 16 μM.

In subsequent in vitro pharmacological evaluations, cells were generally assigned to the following groups: (1) control group, in which cells were cultured under normal conditions without any treatment; (2) model group, in which BV2 and bEnd.3 cells were stimulated with Aβ_1–42_ and LPS; (3) ICT/TS IIA group, in which modeled cells were treated with a combination of free ICT and TS IIA; (4) PBIT@NPs group, in which modeled cells were treated with PBIT@NPs; and (5) KPBIT@NPs group, in which modeled cells were treated with KPBIT@NPs. In some experiments, an additional KPB@NPs group was included as a blank nanocarrier control.

For pathway-validation experiments, cells were divided into five groups. The AMPK inhibition experiments included the control, model, KPBIT@NPs, Compound C, and KPBIT@NPs + Compound C groups. The mTOR activation experiments included the control, model, KPBIT@NPs, MHY1485, and KPBIT@NPs + MHY1485 groups. In the corresponding combination-treatment groups, cells were pretreated with Compound C or MHY1485, followed by treatment with KPBIT@NPs.

**7.2 In vivo experiments**

All animal procedures were approved by the Institutional Animal Care and Use Committee (IACUC). Animals were randomly divided into five groups (n = 6 per group): control group (C57BL/6J mice), model group (APP/PS1 transgenic mice), ICT/TS IIA combination group, PBIT@NPs group, and KPBIT@NPs group. Drugs were administered via tail vein injection. TS IIA and ICT were given at doses of 2 mg/kg and 2.6 mg/kg, respectively, based on previous studies and preliminary evaluations [3–5]. The control and model groups received saline, while treatment groups received the corresponding formulations at a volume of 100 μL every two days for 28 days.

**8 Cell uptake study**

bEnd.3 and BV2 cells were seeded into 48-well plates at a density of 1.2 × 10^4^ cells per well and cultured under standard conditions. After cell attachment, cells in the model and treatment groups were subjected to AD-like stimulation as described above, while the control group received no treatment. The cells were divided into four groups: control group, PBC@NPs group, KPBC@NPs group, and KPBC@NPs + FPS-ZM1 group. For the RAGE blocking group, cells were pretreated with FPS-ZM1 (1 μM) for 30 min at 37°C prior to nanoparticle incubation. Fluorescently labeled PBC@NPs and KPBC@NPs were then added and incubated with the cells for 2 h at 37°C. After incubation, the culture medium was removed, and cells were washed twice with PBS to remove excess nanoparticles. Cells were subsequently stained with Hoechst 33342 (100 μL per well) at room temperature for 15 min in the dark. After staining, cells were washed twice with PBS and observed under a fluorescence microscope (Nikon Eclipse E800, Japan). Images were captured to evaluate nanoparticle internalization.

**9 Transwell assay**

An in vitro AD-BBB co-culture model was established using Transwell inserts. bEnd.3 cells were seeded in the upper chamber at a density of 5 × 10^5^ cells/well and cultured under standard conditions for 7 days until a confluent and compact endothelial monolayer was formed. The endothelial monolayer was subsequently exposed to pre-aggregated Aβ_1–42_ (20 μM), prepared by incubation at 37°C for 24 h, together with LPS (2 μg/mL) for 12 h to generate AD-like injured endothelial cells (AD-bEnd.3). BV2 cells were seeded in the lower chamber at a density of 1 × 10^4^ cells/well and subjected to the same treatment with pre-aggregated Aβ_1–42_ (20 μM) and LPS (2 μg/mL) for 12 h to generate AD-like injured microglia (AD-BV2), thereby establishing the in vitro AD-BBB pathological co-culture model. Multiple parallel models were prepared under identical culture and induction conditions and independently assigned to the sodium fluorescein permeability assay and nanoparticle transport assay to avoid interference between the two experiments. Transendothelial electrical resistance (TEER) was measured daily from days 1 to 7 of culture and again after AD induction to assess barrier integrity. Before measurement, the Transwell plates were equilibrated at room temperature for 30 min, and residual medium on the outer surfaces of the inserts was carefully removed. The electrodes of the resistance meter were vertically immersed in the media of the upper and lower chambers without contacting the endothelial monolayer. Three replicate wells were included in each group, and each well was measured three times. Cell-free Transwell inserts served as blank controls, and the blank resistance was subtracted from the measured resistance. The area-corrected TEER was calculated as TEER (Ω·cm^2^) = (R_sample_ − R_blank_) × 0.33 cm^2^. Stabilization of the TEER value at the predefined barrier-formation threshold was considered indicative of an intact endothelial monolayer with well-developed intercellular tight junctions. Sodium fluorescein permeability was evaluated using an independent set of TEER-qualified AD-BBB models. After removal of the culture media, the cells were gently washed twice with sterile PBS. Sodium fluorescein (Na-FLU) solution at an initial concentration of 50 μg/mL was added to the upper chamber at a volume of 100 μL, while 500 μL of serum-free culture medium was added to the lower chamber. The plates were incubated for 60 min at 37°C under light-protected conditions. The medium in the lower chamber was then collected, and its fluorescence intensity was measured using a multimode microplate reader at excitation and emission wavelengths of 492 and 520 nm, respectively. A Na-FLU calibration curve was generated using standard solutions at concentrations of 0, 0.10, 0.50, 1.00, 5.00, and 10.00 μg/mL, and the Na-FLU concentration in the lower chamber was calculated from the resulting regression equation. The apparent permeability coefficient was calculated as P_app_ = Q/(C₀ × A × t), where Q represents the amount of Na-FLU recovered from the lower chamber, C₀ is the initial donor concentration of 50 μg/mL, A is the Transwell membrane area of 0.33 cm^2^, and t is the incubation time of 3600 s. Nanoparticle transport was assessed using separate TEER-qualified AD-BBB models that had not been exposed to Na-FLU. The media in both chambers were removed, and the cells were washed twice with PBS. Fluorescently labeled PBC@NPs or KPBC@NPs were separately added to the upper chamber, followed by incubation for 12 h under standard culture conditions. After incubation, the medium in the lower chamber was removed, and cells in both chambers were washed twice with sterile PBS to eliminate free and non-transported nanoparticles. Cell nuclei were then stained with 100 μL of Hoechst 33342 per well for 15 min at room temperature in the dark. After washing with PBS to remove residual dye, fluorescence images were acquired using a Nikon Eclipse E800 fluorescence microscope (Nikon, Japan), and nanoparticle transport across the endothelial barrier and cellular distribution were qualitatively evaluated. All experiments were performed using three replicate wells per group (n = 3).

**10 In vivo targeting evaluation**

Free DIR, PBD@NPs, and KPBD@NPs were administered via tail vein injection at a dose equivalent to 2 μg DIR per mouse. Mice were anesthetized by inhalation of isoflurane, and in vivo fluorescence images were acquired over a time period of 1–72 h using an in vivo imaging system (IVScope 8200, Clinx Science Instruments Co., Ltd.). For pharmacokinetic analysis, rats were intravenously injected with the same formulations at an equivalent DIR dose. Blood samples were collected from the retro-orbital sinus at predetermined time points within 1–72 h post-injection. The collected samples were transferred into a 96-well plate for fluorescence imaging analysis. At the endpoint, mice were euthanized, and major organs including the heart, liver, spleen, lungs, kidneys, and brain were harvested. Ex vivo fluorescence imaging was performed to evaluate the biodistribution and brain-targeting efficiency of the nanoparticles. Fluorescence intensity was quantitatively analyzed using region-of-interest (ROI) analysis.

**11 Cell viability assay**

HT22, bEnd.3, and BV2 cells were seeded at a density of 5 × 10^4^ cells/well in 96-well plates. After cell attachment, all groups except the control group were subjected to modeling treatments, followed by incubation with ICT/TS IIA, PBIT@NPs, or KPBIT@NPs for 12 h. Cell viability was evaluated using a CCK-8 assay, and absorbance was measured at 450 nm using a microplate reader. Cell viability (%) was calculated according to the following formula:

*Cell Viability* (%) = $\frac{\text{OD}_{\text{Treatment group}}\text{−}\text{OD}_{\text{Control}\text{ }\text{well}}}{\text{OD}_{\text{Control}\text{ group}}\text{−}\text{OD}_{\text{Control}\text{ }\text{well}}}$×100%

**12 Drug combination synergy analysis**

BV2 cells were seeded into 96-well plates at a density of 1 × 10^4^ cells per well and cultured at 37°C with 5% CO_2_. Cells were divided into control and experimental groups. After cell adhesion, Aβ_1-42_ + LPS were added to all groups except the control group and incubated for 12 h to establish an inflammatory model. Subsequently, ICT, 0–40 μM and TS IIA, 0–50 μM were co-administered to BV2 cells using a checkerboard design and incubated for an additional 12 h. Cell viability was assessed using a CCK-8 assay, and the data were uploaded to the SynergyFinder platform (https://synergyfinder.org/) to evaluate the synergistic effects of the two drugs using the HSA model.

**13 Anti-inflammatory and antioxidant assays**

The levels of pro-inflammatory cytokines (IL-6, TNF-α, and IL-1β) and anti-inflammatory markers (Arg-1 and IL-10) in BV2 cells were determined using commercial enzyme-linked immunosorbent assay (ELISA) kits according to the manufacturers’ instructions. Oxidative stress-related indicators, including malondialdehyde (MDA), glutathione (GSH), and catalase (CAT), were measured using corresponding assay kits following the standard protocols provided by the manufacturers.

**14 Cellular immunofluorescence assay**

BV2 cells were seeded into 48-well plates at a density of 1.2 × 10^4^ cells/well. After reaching appropriate confluence, AD-BV2 models were established in all groups except the control group. Subsequently, cells were treated with the indicated drugs and incubated for an additional 12 h. Following treatment, the culture medium was removed, and cells were washed twice with PBS. Cells were then fixed with 4% paraformaldehyde for 30 min at room temperature and washed twice with PBS. Permeabilization was performed using 1% Triton X-100 for 30 min, followed by blocking with 5% bovine serum albumin (BSA) for 20 min. Cells were incubated with primary antibodies at 4°C overnight. After incubation, primary antibodies were removed, and cells were washed twice with PBS. Subsequently, appropriate fluorescent secondary antibodies (FITC/CY3) were added and incubated for 1 h at room temperature in the dark. Nuclei were stained with Hoechst dye for 15 min, followed by two PBS washes. Finally, fluorescence images were captured using a fluorescence microscope.

**15 JC-1 mitochondrial membrane potential assay**

BV2 cells were seeded into 48-well plates at a density of 1.5 × 10^4^ cells/well and cultured at 37°C in a humidified incubator with 5% CO_2_. After reaching appropriate confluence, AD-BV2 models were established in all groups except the control group. Cells were then treated with the indicated drugs and incubated for an additional 12 h. Following treatment, the culture medium was removed, and cells were gently washed once with PBS. JC-1 staining solution (100 μL per well), prepared according to the manufacturer’s instructions, was added, and cells were incubated at 37°C in the dark for 30 min. After staining, cells were washed twice with PBS. Fluorescence images were captured using a fluorescence microscope, and mitochondrial membrane potential was evaluated based on the ratio of red to green fluorescence intensity.

**16 Flow cytometric analysis of marker expression**

BV2 cells were seeded into 48-well plates at a density of 1.2 × 10^4^ cells/well. After reaching appropriate confluence, AD-BV2 models were established in all groups except the control group. Cells were then treated with drugs and incubated for 12 h. After treatment, cells were washed twice with PBS, detached using trypsin, and collected into centrifuge tubes. Cells were centrifuged and resuspended in PBS. Subsequently, cells were fixed with 4% paraformaldehyde for 30 min at room temperature and washed twice with PBS. Permeabilization was performed using 1% Triton X-100 for 30 min, followed by blocking with 5% BSA for 20 min. Cells were incubated with primary antibodies at 4°C overnight. After washing twice with PBS, cells were incubated with fluorophore-conjugated secondary antibodies (FITC/CY3) for 1 h at room temperature in the dark. The samples were then filtered through a nylon mesh to obtain a single-cell suspension. Finally, fluorescence signals were analyzed using a flow cytometer.

**17 Network pharmacology**

Disease-associated targets for AD were retrieved from the GeneCards, OMIM, and DisGeNET databases using “Alzheimer’s disease” as the keyword. The collected targets were integrated and duplicates were removed. In parallel, the chemical constituents and corresponding targets of *Epimedium* and *Salvia miltiorrhiza* were obtained from the TCMSP database (version 2.3) and relevant literature. Active compounds were screened based on pharmacokinetic parameters, with thresholds set at oral bioavailability (OB) ≥ 30%, drug-likeness (DL) ≥ 0.18, and blood-brain barrier permeability ≥ −0.6. The chemical structures of the selected compounds were validated using the PubChem database, and their canonical SMILES were retrieved. Potential targets were predicted using SwissTargetPrediction. The intersecting targets between compound-related targets and AD-associated targets were imported into the STRING database (version 11.0) to construct a protein–protein interaction (PPI) network, with the species limited to Homo sapiens. The network was visualized using Cytoscape (version 3.8.2). Gene Ontology (GO) enrichment analysis, including biological process (BP), cellular component (CC), and molecular function (MF), as well as KEGG pathway enrichment analysis, were performed for the intersecting targets. The top 20 enriched terms were visualized using the MicroBioinformatics platform.

**18 Molecular docking**

Molecular docking was performed to predict the binding modes of ICT (PubChem CID: 5318980) and TS IIA (PubChem CID: 164676) with AMPK and mTOR. The three-dimensional (3D) structures of ICT and TS IIA were retrieved from the PubChem database in SDF format. Prior to docking, the dominant protonation and tautomeric states of both ligands at physiological pH were checked. Ligand geometries were energy-minimized using ChemBio3D Ultra 22.2.0 under the MM2 force field until the root-mean-square (RMS) gradient was less than 0.001 kcal mol^−1^ Å^−1^. The optimized structures were saved in MOL format and imported into AutoDockTools 1.5.7 for ligand preparation. Polar hydrogen atoms were added, Gasteiger partial charges were assigned, non-polar hydrogens were merged, and rotatable bonds were explicitly defined. The prepared ligands were then saved in PDBQT format.

The crystal structures of the target proteins were obtained from the RCSB Protein Data Bank. The FKBP–rapamycin-binding domain of human mTOR, also referred to as the FRB domain, was selected as the mTOR docking model (PDB ID: 1AUE; X-ray diffraction; resolution, 2.33 Å). Because 1AUE represents the isolated FRB domain rather than the full-length mTOR protein, it was used specifically to evaluate ligand binding within the rapamycin-sensitive FRB regulatory pocket. For AMPK, the full-length human AMPK heterotrimer co-crystallized with the benzimidazole activator 991 was selected (PDB ID: 4CFE; X-ray diffraction; resolution, 3.02 Å). One representative biological assembly of AMPK was used for docking, consisting of the α2, β1, and γ1 subunits. The AMPK docking site was defined around the co-crystallized activator 991-binding pocket, corresponding to the allosteric drug and metabolite site located between the kinase domain of the α subunit and the carbohydrate-binding module of the β subunit.

Protein structures were prepared using PyMOL 3.0.3 and AutoDockTools 1.5.7. For the mTOR FRB domain, one representative FRB chain was retained for docking. For AMPK, one complete heterotrimeric assembly was retained. Crystallographic water molecules and non-essential heteroatoms were removed. Co-crystallized ligands located in the docking pocket were removed before docking to avoid steric interference. For AMPK, the co-crystallized activator 991 was removed to define the ligand-binding cavity, while structurally relevant nucleotide molecules outside the docking box were retained only when they did not overlap with the ligand search space. Missing residues and side chains were inspected before docking; residues absent from regions distal to the defined binding pockets were not modeled, whereas no missing residue directly lining the selected docking cavities was found to interfere with ligand placement. Hydrogen atoms were added, Kollman united-atom charges and AutoDock atom types were assigned, and the final receptor structures were saved in PDBQT format. During docking, receptors were treated as rigid bodies, whereas ligands were treated as flexible molecules.

Docking sites were defined based on co-crystallized ligand positions and literature-reported functional residues. For the mTOR FRB domain, the search space was centered on the rapamycin-sensitive FRB pocket, which includes residues reported to participate in ligand or substrate recognition, such as Leu2031, Ser2035, Phe2039, Trp2101, Tyr2105, and Phe2108. The grid box for mTOR was centered at x = 19.911, y = 3.766, and z = 30.802 Å, with dimensions of 42 × 40 × 66 Å. For AMPK, the search space was centered on the activator 991-binding allosteric pocket. The grid box for AMPK was centered at x = 508.362, y = 45.425, and z = 1013.980 Å, with dimensions of 118 × 126 × 110 Å. The search-box center and size were specified in Å according to the requirements of AutoDock Vina. Because both targets contained structurally defined ligand-binding regions, focused or site-directed docking was performed rather than unrestricted blind docking.

Molecular docking simulations were conducted using AutoDock Vina. For each ligand–receptor system, including ICT–AMPK, ICT–mTOR, TS IIA–AMPK, and TS IIA–mTOR, the maximum number of output binding modes was set to 20, the energy range was set to 5 kcal mol^−1^, and the exhaustiveness parameter was set to 32. To improve reproducibility and assess pose stability, each docking system was subjected to three independent docking runs using predefined random seeds of 202401, 202402, and 202403. The docking pose with the lowest predicted binding affinity was not selected solely on the basis of docking score; instead, the final representative pose was chosen according to the combined criteria of low binding energy, localization within the predefined binding pocket, absence of severe steric clashes, and a geometrically plausible orientation consistent with known pocket residues.

Docking poses were ranked according to the binding affinity scores predicted by the AutoDock Vina scoring function. The top-ranked conformations were visualized and analyzed using PyMOL 3.0.3. Protein–ligand interactions were evaluated by measuring hydrogen bonds, hydrophobic contacts, π–π stacking, electrostatic interactions, and the involvement of key amino acid residues within the binding pocket. Hydrogen bonds were identified based on donor–acceptor distance and geometric orientation, whereas hydrophobic and aromatic interactions were assigned by visual inspection of ligand–residue proximity and orientation.

The docking protocol was validated by re-docking native or reference ligands into their corresponding binding sites when an experimentally defined ligand pose was available. For AMPK, the co-crystallized activator 991 was removed from 4CFE and re-docked into its original allosteric binding pocket. The heavy-atom root-mean-square deviation (RMSD) between the predicted and crystallographic poses was calculated after receptor alignment. An RMSD value below 2.0 Å was considered indicative of an acceptable reproduction of the experimentally observed binding mode. Because the selected mTOR structure 1AUE contains the isolated FRB domain without a co-crystallized small-molecule ligand in the docking pocket, the FRB docking region was further verified by comparison with reported rapamycin-sensitive FRB pocket residues and by ensuring that the docking grid fully covered the canonical FRB ligand-recognition surface. Final docking results were reported as predicted binding affinity values in kcal mol^−1^ together with the corresponding binding poses and key protein–ligand interactions.

**19 Western blot**

Total proteins were extracted from brain tissues and BV2 cells in each group, and protein concentrations were determined using a BCA protein assay kit. Protein samples were mixed with loading buffer and denatured at 95°C for 5 min. Equal amounts of protein (20 μg) were separated by SDS-PAGE (10% gels) and subsequently transferred onto PVDF membranes. The membranes were washed three times with TBST and blocked with 5% skim milk for 1 h at room temperature. The membranes were then incubated overnight at 4°C with primary antibodies against p-AMPK, AMPK, p-mTOR, mTOR, HIF-1α, p-PDHE1α, PDHE1α, PFKFB3, CD86, CD206, and β-actin (dilution 1:1000). After washing three times with TBST, the membranes were incubated with HRP-conjugated secondary antibodies (dilution 1:1000) for 1 h at room temperature. Protein bands were visualized using BeyoECL Plus chemiluminescence reagent and detected with a gel imaging system. Relative protein expression levels were quantified by densitometric analysis using ImageJ software, with β-actin as the internal control.

**20 Detection of metabolic changes in BV2 cells**

**20.1 ECAR assay**

BV2 cells were seeded into a Seahorse XF96 cell culture plate at a density of 1 × 10^4^ cells per well and incubated at 37°C in 5% CO_2_. Cells were divided into control and experimental groups. After attachment, experimental groups were subjected to AD modeling using LPS + Aβ to establish AD-BV2 cells, followed by treatment with ICT/TS IIA, PBIT@NPs, or KPBIT@NPs for 12 h. The Seahorse XF sensor cartridge was hydrated by adding 200 μL sterile water to each well and incubating overnight at 37°C. The following day, water was replaced with XF calibrant, and the cartridge was incubated in a CO_2_-free incubator at 37°C for 60 min. For the assay, Seahorse XF DMEM (pH 7.4, without phenol red) supplemented with glutamine was used as the assay medium. Glucose (100 mM), oligomycin (100 μM), and 2-deoxyglucose (2-DG, 500 mM) stock solutions were prepared. The final working concentrations were 10 mM glucose, 1 μM oligomycin, and 50 mM 2-DG. Cells were washed twice with assay medium and incubated in 180 μL assay medium per well at 37°C in a CO_2_-free incubator for 1 h prior to measurement. Subsequently, 20 μL glucose, 22 μL oligomycin, and 25 μL 2-DG were loaded into ports A, B, and C, respectively. The sensor cartridge was then placed onto the cell plate, and ECAR was measured using a Seahorse XF Analyzer.

**20.2 OCR assay**

OCR measurements were performed similarly to the ECAR assay. Oligomycin (100 μM), FCCP (100 μM), and rotenone/antimycin A (Rot/AA, 50 μM stock) were prepared. The final working concentrations were 1.5 μM oligomycin, 1.5 μM FCCP, and 0.5 μM Rot/AA. Cells were washed and equilibrated in assay medium as described above. Drugs were loaded into ports as follows: oligomycin (port A), FCCP (port B), and Rot/AA (port C). OCR was measured using the Seahorse XF Analyzer according to the manufacturer’s instructions.

**21 Effects of BV2 cell-conditioned medium on HT22 neuronal cells**

**21.1 Preparation of conditioned medium**

BV2 cells were seeded at a density of 3 × 10^6^ cells per well in six-well plates and cultured at 37°C in a humidified incubator with 5% CO_2_. After reaching approximately 70-80% confluence, cells in the control group were maintained under normal culture conditions without Aβ_1-42_, LPS, or drug treatment, and the collected supernatant was used as control conditioned medium (Ctrl-CM). Cells in the model group were treated with Aβ_1-42_ and LPS, whereas cells in the treatment groups were exposed to Aβ_1-42_, LPS, and the indicated formulations for 12 h. The medium was then replaced with fresh complete medium, and cells were further incubated for an additional 12 h. Subsequently, culture supernatants from each group were collected and centrifuged to remove cell debris, and the resulting supernatants were used as conditioned media for subsequent experiments.

**21.2 Calcein-AM/PI**

HT22 cells were seeded at a density of 1 × 10^4^ cells per well in 96-well plates and cultured under standard conditions. After reaching appropriate confluence, cells (except for the control group) were subjected to experimental treatments. Following drug administration for 12 h, cells were washed once with PBS and incubated with staining solution containing Calcein-AM and propidium iodide (PI) for 30 min at 37°C in the dark. Fluorescence images were captured using a fluorescence microscope, and cell viability was quantified using ImageJ software based on standardized thresholding methods. The ratio of PI-positive cells to total cells was calculated to determine cell death rates.

**21.3 Reactive oxygen species (ROS) assay**

BV2 or HT22 cells were seeded at a density of 1.5 × 10^4^ cells per well in 48-well plates. After treatment, cells were washed once with serum-free medium and incubated with DCFH-DA probe at 37°C for 20 min in the dark. After incubation, cells were washed to remove excess probe, and fluorescence images were acquired immediately using a fluorescence microscope. Intracellular ROS levels were quantified by measuring DCF fluorescence intensity using ImageJ software.

**22 In vivo pharmacodynamic evaluation**

For the in vivo therapeutic study, mice were allocated to five experimental groups, with six biologically independent mice per group: (1) wild-type control, (2) untreated APP/PS1 model, (3) APP/PS1 mice treated with free ICT + TS IIA, (4) APP/PS1 mice treated with PBIT@NPs, and (5) APP/PS1 mice treated with KPBIT@NPs. The wild-type group was included to establish the physiological baseline, whereas the untreated APP/PS1 group served as the disease control. The free ICT + TS IIA group was used to evaluate the therapeutic effects of the unencapsulated drug combination. PBIT@NPs, which lacked KLVFFAED functionalization, served as the non-targeted nanoparticle control to assess the contribution of KLVFFAED-mediated blood–brain barrier penetration and activated-microglia targeting. KPBIT@NPs represented the complete multifunctional formulation. The sample size was selected based on the expected variability of behavioral outcomes in the APP/PS1 model and the principle of minimizing animal use.

**22.1 Morris water maze (MWM) test**

After 28 days of drug treatment, the Morris water maze test was conducted while drug administration was continued. The experiment lasted for 6 consecutive days. During the first 5 days (acquisition phase), mice were released from randomly assigned quadrants, and each trial lasted 60 s. The swimming trajectory and escape latency to locate the hidden platform were recorded. On day 6 (probe trial), the platform was removed, and mice were released from the quadrant opposite to the former platform location. The escape latency, time spent in the target quadrant, and number of crossings over the former platform location were recorded. The therapeutic effects of each treatment group were evaluated based on these behavioral parameters (n = 6 biologically independent mice per group).

**22.2 Nest-building test**

Following the MWM test, the nest-building assay was performed with continuous drug administration. Each mouse was individually housed in a cage containing five pieces of cotton (5 cm × 5 cm). Nesting behavior was recorded by photographing the cages at predetermined time points over 24 h. The degree of cotton shredding and nest construction was evaluated according to a previously reported scoring system to assess cognitive and behavioral functions across groups (n = 6 biologically independent mice per group).

**22.3 Measurement of inflammatory factors and mitochondrial function**

The levels of IL-1β, TNF-α, IL-10, and Arg-1 in brain tissue were measured using commercially available assay kits according to the manufacturers’ instructions. Mitochondrial function was assessed using mitochondrial-specific assay kits following standard protocols.

**22.4 Histological and immunostaining analysis**

Hematoxylin and eosin (H&E) staining and Nissl staining were performed to evaluate pathological damage and neuronal injury in brain tissue. Immunohistochemistry was used to detect the expression of GFAP (astrocyte marker), IBA-1 (microglial marker), and Aβ. Immunofluorescence staining was conducted to assess NeuN expression and the co-localization of IBA-1/CD86 and IBA-1/CD206 in brain tissue.

Mitochondrial ultrastructure was evaluated by transmission electron microscopy (Hitachi HT7800, Japan), whereas neuronal dendritic morphology was assessed by Golgi staining. Representative images from each group were collected and analyzed.

**23 In vivo biosafety evaluation**

After administration to mice in the blank and KPBIT@NPs groups, routine hematological parameters, including red blood cells (RBCs), white blood cells (WBCs), lymphocytes (LYM), hemoglobin (HGB), and platelets (PLTs), were analyzed. Commercial ELISA kits were used to detect the levels of inflammatory factors in mouse peripheral blood. The contents of pro-inflammatory factors IL-1β, TNF-α and anti-inflammatory factor IL-10 in peripheral blood were strictly determined in accordance with the standard operating procedures provided by the kit instructions to evaluate the systemic inflammatory response induced by the nanoparticles. Major organs, including the heart, liver, spleen, lung, and kidney, were harvested, fixed in 4% paraformaldehyde, embedded in paraffin, sectioned, and stained with hematoxylin and eosin (H&E) for histopathological evaluation.

**24 Blood compatibility evaluation**

The hemolytic activity of the nanoparticles was evaluated to assess their blood compatibility. Red blood cells (RBCs) were washed with PBS and resuspended to obtain a 2% (v/v) RBC suspension. Equal volumes of nanoparticle suspensions at different concentrations (KPBIT@NPs) were incubated with the RBC suspension at 37°C for 2 h. Deionized water and physiological saline were used as positive and negative controls, respectively. After incubation, the samples were centrifuged to remove intact RBCs, and the absorbance of the supernatant was measured at 540 nm. The hemolysis rate was calculated as follows:

*Hemolysis rate* (%) = $\frac{\text{A}_{\text{Sample group}}\text{−}\text{A}_{\text{Negative control group}}}{\text{A}_{\text{Posi}\text{tive control group}}\text{−}\text{A}_{\text{Negative control group}}}$×100%

**25 Statistical analysis**

Statistical analyses were performed using GraphPad Prism 10 (GraphPad Software, Boston, MA, USA). Unless otherwise specified, all quantitative data are presented as the mean ± standard deviation (SD). The sample size (n) in this study indicates biological replicates, referring to independent animals for in vivo experiments or independently prepared cell cultures for in vitro experiments, and does not represent technical replicates.

For comparisons between two independent groups, an unpaired two-tailed Student’s *t*-test was used. For comparisons among three or more experimental groups, one-way analysis of variance (ANOVA) followed by Tukey’s multiple-comparisons test was performed. Repeatedly measured behavioral data collected from the same animals over time, including the acquisition-phase latency of the Morris water maze and time-dependent nest-building performance, were analyzed using two-way repeated-measures ANOVA, with treatment group as the between-subject factor and time as the within-subject factor, followed by Tukey’s post hoc multiple comparisons. Single-time-point behavioral indices, including the probe-trial parameters of the Morris water maze, were analyzed using one-way ANOVA with Tukey’s multiple-comparisons test.

Detailed sample sizes for each dataset are explicitly stated in the corresponding figure legends. A *P* value less than 0.05 was considered statistically significant. Statistical significance levels were defined as: ^*^*P* < 0.05, ^**^*P* < 0.01, ^***^*P* < 0.001,, ^****^*P* < 0.0001 and ns (not significant, *P* ≥ 0.05).

**References**

[1] H.Y. Nam, J.H. Nam, G. Yoon, J.Y. Lee, Y. Nam, H.J. Kang, H.J. Cho, J. Kim, H.S. Hoe, Ibrutinib suppresses LPS-induced neuroinflammatory responses in BV2 microglial cells and wild-type mice, J Neuroinflammation 15(1) (2018) 271.

[2] X. Zhao, J. Sun, L. Xiong, L. She, L. Li, H. Tang, Y. Zeng, F. Chen, X. Han, S. Ye, W. Wang, X. Wang, G. Liang, beta-amyloid binds to microglia Dectin-1 to induce inflammatory response in the pathogenesis of Alzheimer's disease, Int J Biol Sci 19(10) (2023) 3249–3265.

[3] Z. Li, Z. Li, B. Wang, J. Liu, Influence of release rate, dose and co-administration on pharmacokinetics, pharmacodynamics and PK-PD relationship of tanshinone IIA and tanshinol, Eur J Pharm Sci 168 (2022) 106042.

[4] X.M. Zhang, J. Ma, Y. Sun, B.Q. Yu, Z.M. Jiao, D. Wang, M.Y. Yu, J.Y. Li, J. Fu, Tanshinone IIA promotes the differentiation of bone marrow mesenchymal stem cells into neuronal-like cells in a spinal cord injury model, J Transl Med 16(1) (2018) 193.

[5] L.H. Zhou, Q. Hu, H. Sui, S.J. Ci, Y. Wang, X. Liu, N.N. Liu, P.H. Yin, J.M. Qin, Q. Li, Tanshinone II--a inhibits angiogenesis through down regulation of COX-2 in human colorectal cancer, Asian Pac J Cancer Prev 13(9) (2012) 4453–8.
